# Repurposing of Chemokine Antagonists for Combined Phase‐Resolved Spinal Cord Injury Treatment

**DOI:** 10.1002/advs.202516569

**Published:** 2025-10-28

**Authors:** Alexey A. Belogurov, Georgii B. Telegin, Aleksandr S. Chernov, Anna A. Kudriaeva, Alexey N. Minakov, Maksim V. Rodionov, Vitaly A. Kazakov, Viktor A. Palikov, Yuri M. Poluektov, Rustam H. Ziganshin, Daria A. Orlova, Alena S. Evpak, Daniil A. Barsuk, Fedor A. Mesheryakov, Aldo Spallone, Dmitry S. Asyutin, Igor N. Pronin, Nikolay A. Konovalov, Dmitri Yu Usachev, Alexander G. Gabibov, Alexander N. Konovalov

**Affiliations:** ^1^ Shemyakin and Ovchinnikov Institute of Bioorganic Chemistry Russian Academy of Sciences Moscow 117997 Russia; ^2^ Department of Biological Chemistry Russian University of Medicine Ministry of Health of Russian Federation Moscow 127473 Russia; ^3^ Branch of Shemyakin and Ovchinnikov Institute of Bioorganic Chemistry Russian Academy of Sciences Pushchino 142290 Russia; ^4^ Medical Radiological Research Center (MRRC) named after A.F. Tsyb Branch of the National Medical Radiological Research Center Ministry of Health of Russian Federation Obninsk 249031 Russia; ^5^ N.N. Burdenko National Scientific and Practical Center for Neurosurgery Ministry of Health of Russian Federation Moscow 125047 Russia; ^6^ Department of Clinical Neurosciences NCL‐Neuromed Institute of Neurosciences Rome 00178 Italy

**Keywords:** chemokines, cytokines, mogamulizumab, spinal cord injury, treatment

## Abstract

Spinal cord injury (SCI) is a medical challenge that results in the formation of a glial scar preventing recovery of axonal conductivity. Cytokines and chemokines significantly affect the pathogenesis of SCI and represent important targets for therapeutic intervention. Here, dozens of cytokines and chemokines are dynamically monitored in plasma, cerebrospinal fluid, and injury site released in response to experimental SCI conducted in two rodent strains and patients undergoing surgical removal of intramedullary tumors. Dataset comprising 6,172 cytokine/chemokine values across 8 time points suggests that SCI in mammals is accompanied by a massive cytokine storm in cerebrospinal fluid, mainly driven by CXCL1, IL‐6, and CCL2‐5. Sub‐acute phase is mostly associated with IL‐2, IL‐7, CCL22 and CX3CL1, whereas TNFα and IL17α permanently persists in CNS for even weeks following SCI. The effects of mogamulizumab and chemical antagonists of C‐C/C‐X‐C chemokine receptors TAK‐799, SB225002, and MK‐7123 on SCI recovery in rodents are further estimated. Here blockade of CCR5 and CXCR1/2 chemokine receptors is shown beneficial for amelioration of acute SCI, whereas anti‐CCR4 antibody mogamulizumab readily prevents secondary inflammation in the injured area. Summarizing, the current report claims for a novel combined time‐resolved therapeutic modality in SCI treatment, which supports feasibility and motivates off‐label clinical evaluation in appropriate cohorts.

## Graphical abstract

Spinal cord injury (SCI) in mammals is accompanied by a massive cytokine storm in cerebrospinal fluid, mainly driven by CXCL1, IL‐6, and CCL2‐5. Sub‐acute phase is mostly associated with IL‐2, IL‐7, CCL22, and CX3CL1, whereas TNFα and IL17α permanently persists in CNS even weeks following SCI. Time‐related combined therapy by CXCR1/2 or ‐CCR5 chemical antagonists, mogamulizumab and infliximab, might ameliorate the evolution of SCI sequelae during the acute, sub‐acute, and chronic phases, respectively.

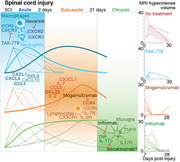

## 1 Introduction

Spinal cord injury (SCI) is a major challenge for modern medicine and a significant problem for society. It may affect up to 6 persons per 100 000 inhabitants annually.^[^ ^1^ ^]^ SCI begins with a sudden, traumatic blow to the spine, with either fracturing or dislocating the vertebral bodies. In the immediate aftermath of trauma, ischemic damage,^[^ ^2^ ^]^ oxidative stress,^[^ ^3^ ^]^ edema.^[^ ^4^ ^]^ and glutamate excitotoxicity.^[^ ^5^ ^]^ are initiated. Subsequently, an inflammatory response occurs at and around the injured area.^[^ ^6^ ^]^ Primary tissue damage is followed by a secondary phase of tissue degeneration that can take several weeks to resolve. This is manifested by glial cell recruitment and proliferation,^[^ ^7^, ^8^ ^]^ infiltration of myeloid cells,^[^ ^9^ ^]^ activation of apoptotic pathways.^[^ ^10^ ^]^ and hypercytokinesis.^[^ ^11^ ^]^ in the damaged spinal cord tissue.

Historically, experimental treatment of SCI started with the use of high‐dose synthetic glucocorticoids.^[^ ^12^ ^]^ However, after almost half of century, current clinical SCI treatment options are extremely limited and based on systemic immunosuppression and surgical decompression. Generally, nowadays there are three basic directions in the search for SCI management, including decreasing of inflammatory process in the damaged area, inducing of restoration of spinal conductivity, and physical bypassing of the injured site. Recently, short‐term inhibition of the fibrinolytic system,^[^ ^13^ ^]^ preserving histone acetylation.^[^ ^14^, ^15^, ^16^ ^]^ and neurotransmitter–agonist conjugates,^[^ ^17^ ^]^ suppression and c‐Jun N‐terminal kinase pathway phosphorylation by salidroside,^[^ ^18^ ^]^ blocking ion channels,^[^ ^19^, ^20^, ^21^ ^]^ carbon monoxide.^[^ ^22^ ^]^ and genetically programmed protein hydrogel.^[^ ^23^ ^]^ delivery, spinal cord.^[^ ^24^, ^25^, ^26^ ^]^ or cortical.^[^ ^27^ ^]^ electrical stimulation, transplantation of neural stem cell grafts.^[^ ^28^, ^29^, ^30^ ^]^ and brain–spinal cord interfaces.^[^ ^31^ ^]^ were reported as promising approaches to overcome SCI.

Hundreds of ongoing clinical trials.^[^ ^32^ ^]^ are evaluating mostly technologies,^[^ ^25^, ^33^ ^]^ rehabilitation, drugs.^[^ ^34^, ^35^ ^]^ and cell‐based approaches.^[^ ^36^, ^37^ ^]^ The majority of these techniques are faced with the problem of the impossibility of regeneration of nerve fibers terminally died, not from the initial traumatic impact but further destroyed by secondary damage, caused by overintense inflammation in the injury site. In many cases, it may be beneficial to save remaining functional neurons, thus preserving sufficient nerve conductivity. Among the relevant damaging factors occurring after the acute phase of SCI, the release of cytokines following trauma generally orchestrates the overall disease course.^[^ ^38^, ^39^ ^]^ and final outcomes.^[^ ^40^, ^41^, ^42^ ^]^ During the first 24 h following SCI, the synthesis and release of potent inflammatory mediators is significantly increased at the lesion site and further play an important role in the development of secondary tissue degeneration in both animals and humans. Thus, Gadani et al. showed that release of IL‐33 from damaged oligodendrocytes drives monocyte recruitment during SCI.^[^ ^43^ ^]^ Levels of tumor necrosis factor (TNFα) and neutrophil‐recruiting CXC motif chemokine ligand 1 (CXCL1) are significantly upregulated in Wistar rats with SCI.^[^ ^44^ ^]^ The IL‐6, IL‐8 (GRO/KC in rodents), and CCL2/MCP‐1 were elevated in an injury‐dependent manner in cerebrospinal fluid (CSF) of patients.^[^ ^45^ ^]^ as well as in the experimentally injured spinal cord of rodents.^[^ ^46^ ^]^ Among chemokine (C‐C motif) ligands (CCL), macrophage inflammatory protein 1‐α (CCL3/MIP‐1α) is responsible for secondary injury after SCI,^[^ ^47^ ^]^ level of CCL5/RANTES was remarkably increased in the astrocytes of rat injured spinal cord,^[^ ^48^ ^]^ and level of mRNA coding for CCL4/MIP‐1β is elevated in mice with SCI,^[^ ^49^ ^]^ while CCL4 itself increase transmigration of lymphocytes through model blood brain barrier cell monolayers.^[^ ^50^ ^]^ Therefore, anti‐cytokine/chemokine therapy seems to be potentially effective in SCI treatment.^[^ ^51^ ^]^ Indeed, several reports demonstrated the efficacy of anti‐TNF antibody infliximab,^[^ ^52^, ^53^ ^]^ anti‐IL6R antibody tocilizumab.^[^ ^54^, ^55^ ^]^ and orally available CXCR1/CXCR2 inhibitor reparixin^[^ ^44^ ^]^ for SCI management.

Despite massive data describing the release of pro‐inflammatory mediators during SCI, current data are mosaic with no evident linkage between animal models and humans. Here we therefore focused on the dynamics of cytokine storm during experimental SCI in two rat strains as well as in a group of patients submitted to surgery for intermedullary spinal cord tumors. After validation of major therapeutic targets, we probed the effect of anti‐CCR4 antibody mogamulizumab as well as three low‐weight chemical inhibitors of C‐C and C‐X‐C motif chemokine receptors CCR5/CXCR3 and CXCR1/2. None of these substances have been tested for SCI treatment earlier. We also tested tocilizumab and infliximab to evaluate the general effect of human monoclonal antibodies in rats independent of its neutralizing activity, as both these antibodies do not block cytokine signaling in non‐humanized rodents.^[^ ^56^, ^57^ ^]^ Animals with SCI were monitored in terms of histological changes, magnetic resonance imaging (MRI), behavior, and locomotor tests. Our data suggest that blockade of chemokine receptors is beneficial for the amelioration of the acute SCI phase, and mogamulizumab readily prevents secondary inflammation in the injured area.

## 2 Experimental Section

### 2.1 Sex as a Biological Variable

The study examined male and female patients and male rats because the SCI model was significantly more reproducible in male animals due to its size, which was beneficial for surgery manipulations. As cytokine and chemokine upregulation was several orders of magnitude above the background, and gender‐specific cytokine release was not detected in female patients versus male patients, the findings were expected relevant for both sexes.

### 2.2 Patients

The objective of this study was to evaluate the cytokine profile changes in patients who underwent surgery due to an intramedullary spinal cord tumor (IMSCT) at the N.N. Burdenko Neurosurgical Institute between February and July 2021. The study was approved by the Ethics Committee of N.N. Burdenko Neurosurgical Institute with protocol reference number 01/2021 dated 01/20/2021. Ten patients who met the inclusion criteria (**Table** **1**) were recruited in the study. Two patients were excluded due to the results of the histological study (hemangioblastoma and high‐grade glial tumor, NOS). The demographic data on patients were listed in Table 1. For each patient, an individual registration card was completed, containing the following information: gender, age, tumor size, histological examination data, Karnofsky index before surgery and on the day of discharge, time of CSF and plasma samples collection, the date of the patient's first follow‐up visit (after three months), the presence or absence of imaging studies after three months, and the Karnofsky index after three months. The tumor size was measured on T1‐weighted images with contrast enhancement in accordance to the functional spinal unit (FSU). The length of the lesion was calculated in mm by measuring the most cranial and caudal points of the tumor on the MR‐tomogram, with the greatest contrasting volume of the tumor. The Karnofsky scale was measured by the attending physician. A histological study was conducted in accordance with the criteria set forth in the fourth edition of the World Health Organization (WHO) Classification of Tumors of the Central Nervous System (2016). Following surgery, all patients received a 1000 mg intravenous methylprednisolone drip, followed by an 8 mg intramuscular dexamethasone injection for four days. A gastroprotective regimen with omeprazole was administered. Pain syndrome was managed with non‐steroidal anti‐inflammatory drugs. Routine blood tests were conducted on the second and fifth days after surgery. All patients were allowed out of bed on the second day after surgery, after clinical and instrumental examination of the lower limb veins. From the second day after surgery until discharge, rehabilitation treatment was carried out. Patients were discharged at an average interval of 8.6 days following surgery. No additional surgical procedures or additional therapy were required for these patients.

**Table 1:** Inclusion, non‐inclusion, and exclusion criteria for patients with IMSCT.

Inclusion	Non‐inclusion	Exclusion
Adult patients, according to the legislation of the Russian Federation	Patient declined to subscribe the informed consent	Patients wish
Written informed consent	Inability to perform MRI (pacemaker, metal prostheses, etc.)	Inability to collect plasma samples
IMSCT	Intolerance to contrast agent	Inability to collect a cerebrospinal fluid (CSF) sample
IMSCT on MRI with contrast enhancement	Competing cancer	Tumors other than ependymoma (Grade 2 or 3), as confirmed by histological studies
Presence of indications for surgery^[^ ^58^ ^]^	History of demyelinating, inflammatory disease of the central nervous system (CNS)	Need for revision surgery (for any reason)
Karnofsky scale score 50 and above	Trauma affecting the CNS	Infectious and inflammatory complications
	Confirmed infectious diseases	Death
	Confirmed mental illness	
	Contraindications for the conventional approach for IMSCT surgery^[^ ^59^ ^]^	

### 2.3 Intradural Catheter Installation

The intradural catheter Perifix (B‐Braun) was installed in the operating room in aseptic conditions prior to the surgical procedure due to IMSCT. The patient was positioned on their right side with the hips and knees flexed to the maximum extent possible. Subsequently, after processing the surgical field, the dural sac was punctured with a Tuohy needle at the L3‐L4 or L4‐L5 through the intralaminar space until CSF was obtained. Then the epidural catheter was introduced and fixed to the skin by a silk suture. The distal end of the catheter was affixed to the skin with an aseptic adhesive tape and sealed.

### 2.4 Surgical Removal of Intramedullary Tumors

All patients were prepared for surgical intervention in accordance with the routine of the N.N. Burdenko Neurosurgical Institute. The surgical intervention was conducted under total intravenous anesthesia and artificial lung ventilation. The patients were placed in the prone position, with the head fixed in a Mayfield‐Kees brace (for tumors involving the cervical spine). All patients underwent a resection laminectomy at the access stage, with the volume of laminectomy calculated based on the size of the contrasting portion of the tumor. Subsequently, the dura mater was incised and the edges were sutured to the muscles. The midline was identified under the guidance of neurophysiological monitoring. Myelotomy and the removal of the intramedullary tumor were performed under an operating microscope and neurophysiological monitoring (motor evoked potentials, D‐wave) in accordance with the standards of high‐tech medical care in the Russian Federation and international recommendations on intramedullary tumors' surgical management.^[^ ^59^ ^]^ Following the removal of the tumor, haemostasis was achieved with careful bipolar coagulation and hemostatic gauze. The pial sheath was closed with a single either 5/0 or 6/0 suture. Subsequently, the dura mater was sutured with HR 17. The wound was closed in layers by with separated sutures, without the need for wound drainage.

### 2.5 Human Sample Collection

The blood samples were collected in 4 mL Li‐heparinized test tubes and then centrifuged at 1500 g for 10 min. Subsequently, a portion of the plasma was transferred to another container and frozen at a temperature of −70 °C. The cerebrospinal fluid (CSF) samples were collected via an intradural catheter placed in a sterile single‐use 10 mL syringe, which was subsequently transferred to a tube and frozen at a temperature of −70 °C. Each probe was marked in accordance with the patient ID and the time elapsed since surgery. The time of trauma onset was calculated from the moment of tumor removal. The samples of CSF and plasma were collected before surgery (0), after 8, 12, 24, 48, 72, 120, and 168 h following traumatic injury.

### 2.6 Laboratory Animals

Male SD and DA rats, weighing ≈368 ± 25 and 263 ± 14 g, respectively, were used in the present study. All animals were housed under standard conditions in the Animal Breeding Facility of BIBCh, RAS (the Unique Research Unit Bio‐Model of the IBCh, RAS; the Bioresource Collection – Collection of SPF‐Laboratory Rodents for Fundamental, Biomedical and Pharmacological Studies). All manipulations with the animals were approved by the IACUC of the BIBCh RAS (protocol no. 913/23 of 24.01.23).

### 2.7 Surgical Approach to Spinal Trauma

The experimental technique has been reported in detail previously.^[^ ^42^, ^60^ ^]^ Briefly, animals were anesthetized by 3% isoflurane to keep cardio, respiratory function, and body temperature constant. Then skin incision was performed in the lumbar midline, spinous process Th13 was carefully removed, and the underline spinal Jura. Exposed under an operating table microscope (Optica, Italy) unilateral hemilaminectomy was performed using a 1 mm diamond burr. The controlled spinal cord cryoinjury was performed by 1 min of contact of copper cryo‐conductor supercooled by liquid nitrogen with the spinal cord. In the case of the compression model, SCI was mediated by the same probe under a pressure of 50 g payload. All surgical manipulation was completed under an aseptic condition. Fully careful wound closure. The animals were then allowed to recover.

### 2.8 SCI Treatment by Neutralizing Antibodies and Low Weight Antagonists

Monoclonal antibodies tocilizumab, infliximab (38.5 mg kg^−1^), and mogamulizumab (4 mg kg^−1^) in a volume of 500 µl were i.v. Injected directly after SCI and 5 days later. Dexamethasone, TAK‐779, SB225002, and MK‐7123 in a volume of 200 µl were i.p. injected in a dosage of 2 mg kg^−1^ directly after SCI and further daily for 5 days. All substances were obtained from MedChemExpress, NJ, USA. The 0.9% NaCl solution was used as a placebo.

### 2.9 Magnetic Resonance Imaging

For MRI of the rat spinal cord, a 7T MRS^*^DRYMAG 7017PW magnetic resonance tomograph (MRSolutions, UK) using a universal radiofrequency coil for the rat body was used. During imaging, animals were fixed in a special heated crib (+37 °C), anesthetized with 3% isoflurane in an oxygen flow, and monitored in terms of respiratory rate and gating function. MRI was performed using standard FSE pulse sequences to obtain T_1_‐ and T_2_‐weighted images, including the caudal thoracic and cranial lumbar spinal segments in the zone of interest, in axial projection with a slice thickness of 0.5 mm, with zero slice spacing. Qualitative and quantitative analysis of the obtained images was performed using VivoQuant software (Invicro, UK). Hyperintense and hypointense areas were segmented corresponding to the localization of the lesion area (spinal cord, Th13 level on the right) In semiautomatic mode (by signal level gradation) or in manual mode (manual delineation of the areas). The volume of hyperintense and hypointense areas was measured in mm^3^.

### 2.10 Diffusion Tensor Imaging

MRI tractography was performed on 7T MRS^*^DRYMAG 7017PW magnetic resonance tomograph equipped by a combined spinal cord radiofrequency coil with a surface receiving element “ASSY‐RSA‐2CH 7T” (MRSolutions, UK). The region of interest was defined as the spinal cord at the level of the Th12‐L1 vertebrae. The animal was positioned in a special heated crib (+37 °C), in ventral position with the level of Th13 in the center of the analyzed area, anesthetized with 3% isoflurane in the flow of oxygen; in the process of imaging the respiratory rate was monitored, and correction of image acquisition was performed using the respiratory gating function. The following study parameters were used: EPI‐DTI protocol, number of DW vector directions: 67, flip‐angle: 90, b‐values: 0 and 700, TE: 27 msec, TR: 5000 msec, axial slice orientation, number of slices: 35, slice thickness 0.5 mm, FOV isotropic, 60 mm, number of experiments with averaging: 1, fat‐saturation on, read interleave on. After acquisition, the data were subjected to additional reconstruction using software filters to remove major artifacts (Nyquist ghost correction, frequency drift correction, vertical motion shift). The final data were converted to NIFTI format and further analyzed using DSI Studio software. Preliminary image preparation with manual segmentation and formation of the spinal cord substance mask (without roots) and exclusion of other tissues from quantitative analysis was performed. Further, ROI (regions of interest) volumes of the left and right halves of the spinal cord were formed by manual segmentation. Under standard conditions, nerve tract reconstruction and FA (fractional anisotropy) measurement were performed at 72 points along the main axis of the spinal cord separately for each half of the spinal cord. The obtained data were exported as arrays and graphs. To correct for the uneven signal from the edges of the coil, 8 measurement points (3 from the cranial, 5 from the caudal edge) were excluded from further calculations as points with unacceptably weak signal levels. Assuming that the true FA values of an intact animal do not differ significantly at different levels of the spinal cord from Th12 to L1, correction factors for the remaining edge sites were obtained from the averaged data from the spinal cord of 5 intact rats; these factors were subsequently used to correct measurement artifacts related to the properties of the RF coil in the experimental groups. The resulting FA values were analyzed by graphical representation of the FA index for the left and right halves of the spinal cord, and further FA for each level of the right (injured) half of the spinal cord relative to that of the left (intact) was calculated.

### 2.11 Histological Studies and multiplex array analysis

Rat spinal cord samples in a bone frame corresponding to the length of 3 vertebrae (access vertebra – Th13, 2 neighboring vertebrae: cranial ‐ Th12 and caudal ‐ L1) were fixed in 10% neutral formalin solution for 3‐5 days, intensively rinsed by water, and further decalcified in Trilon B at room temperature for 12–16 days. Next, the specimens were excised to remove soft tissues and to orient the biomaterial for further microtomy in the sagittal plane. The excised fragments were washed in running tap water, dehydrated in ethyl alcohol of ascending concentration, and poured into homogenized paraffin embedding medium Bio Plast plus (Bio‐Optica, Italy). Microtomy of specimens was performed using a rotary microtome with a Microm HM 340E slice transfer system (Germany) in the sagittal (segmental) plane of the spinal cord. Paraffin sections for overview microscopy were stained with hematoxylin and eosin. The fibrous component in the structure of the glial scar was analyzed by Azan trichrome staining kit (Bio‐Optica, Italy). Selective staining of astrocytic glia in the focus of cryodestruction was performed by detection of glial fibrillary acidic protein (GFAP). Rabbit anti‐rat antibodies were used to detect GFAP‐positive cells, and FITC‐labeled goat antibodies were used as a secondary antibody. The stained preparations were encapsulated in Bio Mount synthetic mounting medium (Bio‐Optica, Italy). Histologic preparations were studied using conventional light microscopy on an AxioScope A1 microscope (Carl Zeiss, Germany). Microphotographs of histological preparations were obtained using a high‐resolution Axiocam 305 color camera (Carl Zeiss, Germany), and morphometric measurements were performed using ZEN 2.6 lite software (Carl Zeiss, Germany). Multiplex assays were performed using the Bio‐Plex Pro Rat Cytokine 23‐Plex kit (#12005641, Bio‐Rad, USA) and Milliplex Human Cytokine/Chemokine Magnetic Bead Panel Premixed 41‐Plex kit (HCYTMAG60PMX41BK, Merck, Germany). Sample preparation was accomplished as described previously.^[^ ^42^ ^]^ The multiplex analysis was conducted following the manufacturer's protocol using Bio‐Plex Manager™ 6.0 software (Bio‐Plex 200 System, Bio‐Rad, USA).

### 2.12 Grip Strength Test

Grip strength test was measured using the Grip Strength apparatus (Columbus Instruments, Columbus, OH, USA). The therapeutic dynamometer force in kg (Chatillon DFIS‐10, AMETEK, Inc / Columbus Instruments, Columbus, OH, USA) was used to record muscle strength separately for the right hind and left hind limbs. Values of the grip strength test represent the difference between left and right paws in kilograms.

### 2.13 Assessment of the Locomotor Activity

Two independent researchers assessed of the locomotor functions of the experimental animals by Basso, Beattie, and Bresnahan (BBB) scale in the preoperative period and daily during a 60‐day post‐injury period as described previously.^[^ ^60^ ^]^ To perform the BBB test, each rat was individually placed in an open field with a non‐slip floor where the animal could move freely for 5 min. Movements of the animals in the open field test and their ability to use hind limbs according to the BBB locomotor rating scale ranged from 0 (total absence of movements) to 21 (regular movements).

### 2.14 Spinal Cord Isolation for Proteomic Studies and Western Blotting

Spinal cord was isolated from intact rats and animals with cryoapplication or compression SCI 15 h post injury. Fresh‐frozen collected tissues were stored in liquid nitrogen. Samples were mechanically homogenized in excess of the TBEP buffer (Thermo Fisher Scientific, USA) in a 10:1 (w/w) ratio. Homogenates were vortexed for 15 s and further incubated at 4 °C for 5 min, then centrifuged at 25 000 g for 20 min at 4 °C. Protein concentration in supernatant was 6–8 mg mL^−1^. Western blotting was accomplished with 100 µg of pooled spinal cord lysates subjected to SDS‐PAGE and further transferred to a nitrocellulose membrane. Anti‐Tbx21 (Ab48787, Abcam), anti‐β‐arrestin‐2 (Ab77208, Abcam), anti‐GFAP (Ab10062, Abcam), ECD‐conjugated anti‐CD14 (IM2707U, Beckman Coulter) and Anti‐β‐Actin‐Peroxidase antibody (A3854, Sigma–Aldrich) were used as primary antibodies. For proteomic studies, 20 µg of protein from each sample was precipitated by adding five volumes of cold acetone. Samples were incubated overnight at −20 °C, followed by centrifugation at 25 000 g for 20 min. After that, supernatants were carefully removed, avoiding pellet disruption. The pellets were washed once with cold acetone under the same conditions. To achieve complete dehydration, pellets were dried using a lyophilizer for 15 min before subsequent analysis. Reduction, alkylation, and digestion of the proteins were performed as described previously.^[^ ^61^ ^]^ with minor modifications. Briefly, 20 µl of sodium deoxycholate (SDC) reduction and alkylation buffer pH 8.5 containing 100 mm Tris, 1% (w/v) SDC, 10 mm TCEP, and 20 mm 2‐chloroacetamide, were added to a 20 µg protein sample. The sample was sonicated in an ultrasonic water bath for 1 min, heated at 85 °C for 10 min, cooled to room temperature, and an equal volume of trypsin solution in 100 mM Tris pH 8.5 was added in a 1:50 (w/w) ratio. After overnight digestion at 37 °C, peptides were acidified by 40 µl of 2% trifluoroacetic acid (TFA) mixed with 80 µl of ethyl acetate and loaded on SDB‐RPS StageTips contained two 14‐gauge SDB‐RPS plugs, and the StageTip was centrifuged at 300 g until all solution go through the StageTip (typically 5 min). After washing the StageTips with a 100 µl of 1% TFA/ethyl acetate 1:1 mixture (2 times) and 50 µl of 0.2% TFA, peptides were eluted in a clean tube by 60 µl 50% acetonitrile/5% ammonia mixture using centrifugation at 300 g. The collected material was vacuum‐dried and stored at −80 °C. Before analysis, peptides were dissolved in 2% acetonitrile/0.1% TFA buffer at a concentration of 0.5 µg µl^−1^ and sonicated for 1 min.

### 2.15 DIA‐LC‐MS Analysis

DIA‐LC‐MS analysis was performed as described previously with minor modifications.^[^ ^62^ ^]^ Peptides were loaded to a home‐made trap column 50x0.1 mm, packed with Reprosil‐Pur 200 C18‐AQ 5 µm (Dr. Maisch), in the loading buffer (2% ACN, 98% H_2_O, 0.1% TFA) at 4 µl min^−1^ flow and separated at RT in a home‐packed.^[^ ^63^ ^]^ fused‐silica column 300x0.1 mm packed with Reprosil‐Pur C18‐AQ 1.9 µm (Dr. Maisch) into an emitter prepared with P2000 Laser Puller (Sutter, USA). Reverse‐phase chromatography was performed with an Ultimate 3000 Nano LC System (Thermo Fisher Scientific), which was coupled to the Orbitrap Tribrid Lumos mass spectrometer (Thermo Fisher Scientific) via a nanoelectrospray source (Thermo Fisher Scientific). Water containing 0.1% (v/v) FA was used as mobile phase A and ACN containing 0.1% FA (v/v), 20% (v/v) H_2_O as mobile phase B. Peptides were eluted from the trap column with a linear gradient: 3–6% B for 5 min, 6–35% B for 53 min, 35–60% B for 4 min, 60% B during 6 min, 60–99% B for 0.1 min, 99% B during 7 min, 99–2% B for 0.1 min at a flow rate of 500 nl min^−1^. MS data was collected in DIA mode. In overlapping window DIA‐MS parameters, MS1 spectra were collected in the range of m/z 495‐745 at 15000 resolution to set an AGC target ‐ Standard. MS2 spectra were collected at m/z 200–1800 at 50 000 resolution to set a normalized AGC target of 2000%, a maximum injection time of “auto”, and stepped normalized collision energies of 22, 26, and 30%. The width of the isolation window was set to 4 Da, and overlapping window patterns at m/z 500–740 were used for window placements.

### 2.16 DIA‐NN Data Analysis

Search parameters of DIA‐NN.^[^ ^64^ ^]^ (version 2.2.0) were set as follows: precursor FDR 1%; scan window set to 0; isotopologues and MBR turned on; protein inference at gene level; heuristic protein inference enabled; quantification strategy set to Quant UMS (high precision); neural network classifier single‐pass mode (cross‐validated); mass accuracy at MS1 and MS2 set to both 0. The settings for in silico library generation (Rattus norvegicus ref. proteome UP000002494) were as follows: Trypsin/P with maximum 1 missed cleavage; protein N‐terminal M excision on; Carbamidomethyl on C as fixed modification; oxidation M and Ac(N‐term) as variable modifications; maximum variable modifications 1; peptide length from 7 to 30; precursor charge 1–4; precursor m/z from 300 to 1800; fragment m/z from 200 to 1800.

### 2.17 Study Approval

Patients who underwent surgery due to an intramedullary spinal cord tumor (IMSCT) at the N.N. Burdenko Neurosurgical Institute between February and July 2021 were recruited in this study. Research protocol was approved by the Ethics Committee of N.N. Burdenko Neurosurgical Institute with protocol reference number 01/2021 dated 01/20/2021, and written consent was obtained from each patient. Animal studies complied with the Declaration of Helsinki and were approved by the IACUC of the BIBCh RAS (protocol no. 913/23 of 24.01.23).

### 2.18 Statistics

The statistical analysis was performed by SPSS Statistics 28 software (IBM, Armonk, NY, USA). Tukey's honestly significant difference test (Tukey's HSD) was used to assess the difference between treated and non‐treated animals. Spearman's rank correlation coefficient (r_s_) with two‐tailed p values was used to estimate the correlation between cytokine and chemokine levels and tumor parameters or neurological disfunction. Correlation between human and rat cytokine values was approximated with a power function y = ax^n^. To assess the relationships between expression levels of individual cytokines in the samples, the ordinary least squares method was applied. The coefficient of determination (R^2^) and p‐value were calculated to evaluate the statistical significance. Analyses were conducted in Python (version 3.11) using the scikit‐learn library (version 1.5.0). Based on the resulting correlation coefficients, heatmaps with hierarchical clustering were constructed along both axes.

### 2.19 Clustermap Reconstruction

To identify samples with similar cytokine expression profiles, hierarchical clustering was performed, followed by visualization in the form of heatmaps. Values were pre‐transformed using the log_10_(x) formula to stabilize variance and approximate a normal distribution. Zero and negative values were replaced with the minimum positive value in the data matrix (equal to 1) to avoid errors during logarithmic transformation. Euclidean distance was used as the distance metric, and agglomerative clustering was performed using the average linkage method. Clustering was applied to the protein matrix, while the samples in the heatmaps were ordered by study day and patient ID. Heatmaps were generated in Python (version 3.11) using the seaborn library (version 0.13.2).

### 2.20 PCA and t‐SNE Visualization

To reduce data dimensionality and explore the sample structure, principal component analysis (PCA) and t‐distributed stochastic neighbor embedding (t‐SNE) were applied. Data on CSF level of CCL11/Eotaxin, G‐CSF, CX3CL/Fractalkine, IFNα2, CXCL1/GRO, IL‐10, IL‐1β, IL‐4, IL‐6, IL‐8, CXCL10/IP‐10, CCL2/MCP‐1, CCL3/MIP‐1α, CCL4/MIP‐1β and TNFα were normalized prior to calculations. PCA was used for preliminary linear dimensionality reduction, and t‐SNE for non‐linear projection into a 2D space (parameters: perplexity = 30, learning_rate = 200). Analyses were performed in Python (version 3.11) using the scikit‐learn library (version 1.5.0), and visualizations were generated with seaborn (version 0.13.2).

### 2.21 Differential Expression Analysis and Volcano Plot Reconstruction

Protein expression differences between study groups were assessed using calculated log_2_ fold change (log_2_FC) values and p‐values. Volcano plots were generated in Python (version 3.11) using the Matplotlib library (version 3.8.4). Thresholds for statistical significance were set at |log_2_FC| > 1 and p‐value < 0.05 (equivalent to –log_10_(p) > 1.3). Gene Ontology (GO) functional annotations were retrieved from the Ensembl Genes 114 database and processed in Python (version 3.11) using the pandas library (version 2.2.2).

## 3 Results

### 3.1 Spinal Cord Injury Modelling in Rats

**Figure 1:**
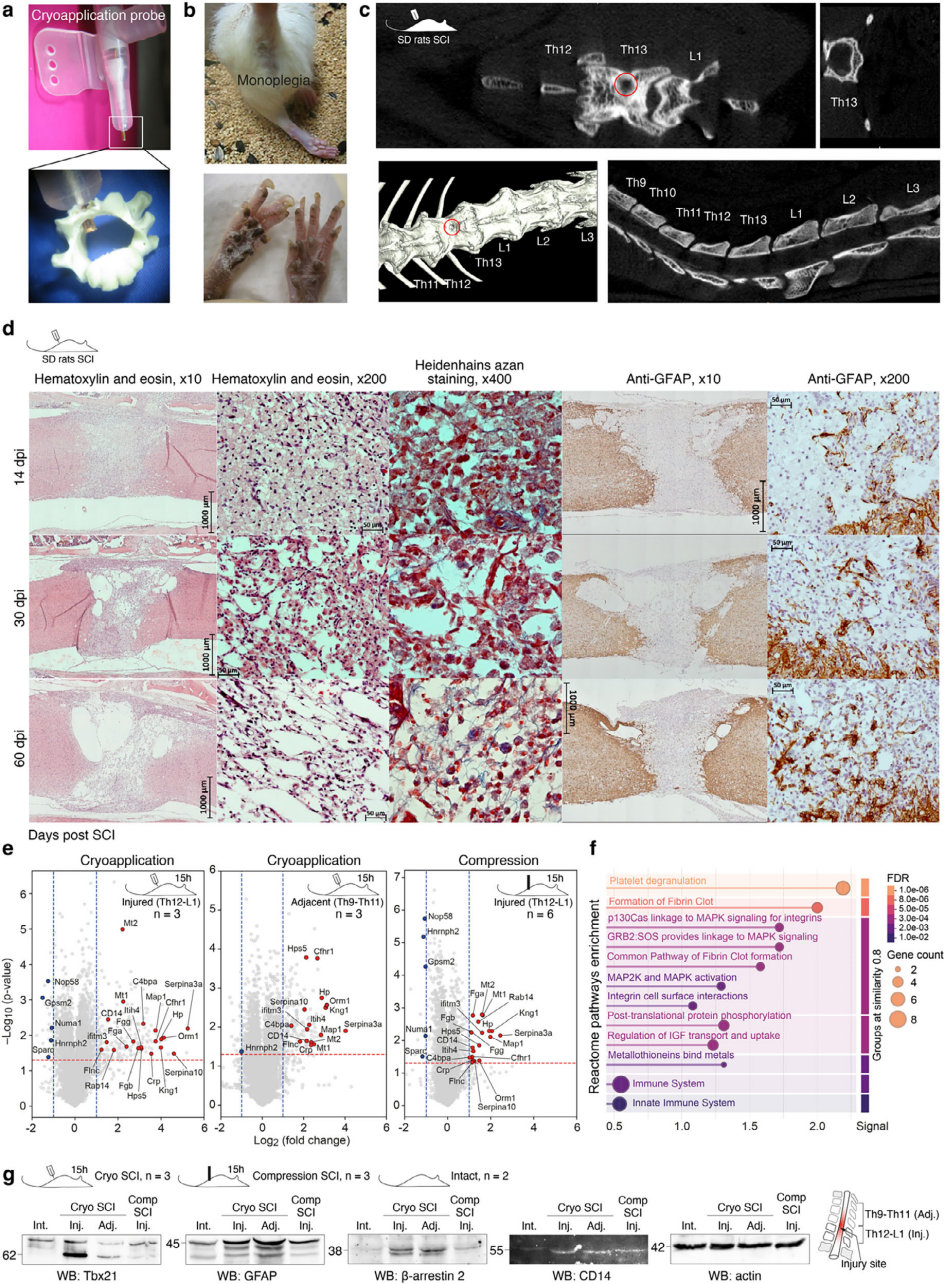
Modelling of spinal cord injury in rats. a) A custom cryoprobe (top) and spinal segment with a drilled hole to access the spinal cord (bottom). b) Representative clinical manifestation of monoplegia in rats with model SCI. c) Computed tomography of the spine and surgical access site to the spinal cord. d) Microscopic examination of spinal cord defect in SD rats, from left to right: (i) panoramic images, hematoxylin and eosin staining; (ii) central part of spinal cord defect, hematoxylin and eosin staining; (iii) configuration, density and distribution of mature collagen fibers in the structure of the glial scar, Heidengain staining with azan; (iv) immunohistochemical detection of GFAP‐positive cells; (v) activation of astrocytic glia in the projection of gray matter of the cranial part of the defect on the border with intact tissue, immunohistochemical detection of GFAP‐positive cells. e) Vulcano plots representing proteomic analysis of intact versus injured spinal cord isolated from rats with cryoinjury and compression SCI models. Shared upregulated proteins are shown in red. f) Activated reactome pathways related to elevated proteins common between cryoinjury and compression SCI models. g) Western blotting analysis for T‐box transcription factor 21 (Tbx21), GFAP, β‐arrestin‐2, and CD14 level in injured (Inj.), adjacent (Adj.), and intact (Int.) spinal cord isolated from rats with cryoapplication (Cryo) and compression (Comp) SCI.

Existing traumatic SCI models provide rather heterogeneous damage of the spinal cord, which very often results in a higher spread of injury rate within the group than between experimental groups itself. These features dramatically limit statistical analysis and yield a binary “yes or no” result of the experimental therapy. In our study, we used a previously developed highly reproducible rodent model of SCI.^[^ ^60^ ^]^ in which the spinal cord is damaged by a copper cryoapplication probe supercooled by liquid nitrogen introduced through a drilled hole using a microsurgical technique in the spinal column (**Figure** **1**a). This methodology results in 85% monoplegia of injured animals (Figure 1b). Computed tomography (CT) was used to confirm the level of access in Th13 (Figure 1c; Video S1, Supporting Information). One section in the sagittal plane with the largest defect area and 2 adjacent sections – above and below the section with the largest defect area – were selected for morphometry of the vertebra Th13 at the level of cryolesion.

During the subacute (3–14 days) and intermediate (21–60 days) observation periods, foci of cryodestruction on serial sagittal sections of the spinal cord were transmural as they covered the entire dorsoventral dimension of the spinal cord. On the 14th day after cryoinjury, increased astroglial reactivity was observed in the cranial and caudal parts of the defect on the border with intact tissue, especially in the projection of dorsal and ventral white matter tubules (Figure 1d). The depth of penetration of GFAP‐positive cells at the border of the defect with the intact tissue was limited to an average of 271 µm (maximum – 428 µm), and the number of GFAP‐positive cells in both cranial and caudal parts of the defect at the border with intact tissue corresponded to an average of 8 cells (maximum – 13) (Table S1, Supporting Information).

On the 30th day of observation, a pronounced progressive reactivity of astrocytic glial cells was observed at the periphery of the defect in the projection of dorsal and ventral tubules of white matter, as well as in the projection of gray matter (Figure 1d). The depth of penetration of GFAP‐positive cells into the structure of the spinal cord defect did not exceed 455 µm (average – 305 µm), and the median count of GFAP‐positive cells both in the cranial and caudal parts of the defect on the border with the intact tissue reached 14 astrocytes (maximum – 27) (Table S1, Supporting Information). Axonal regeneration was hindered by the large cystic cavities formed by this time at the border of the defect with the intact tissue, mainly in the projection of the dorsal tubules of the white matter in both the cranial and caudal parts of the defect (Figure 1d).

At the end of 60 days of observation, glial cells were still located along the edge of the defect (Figure 1d). Immunohistochemical investigation of GFAP‐positive cells revealed that the average depth of its penetration into the structure of the spinal cord defect did not exceed 342 µm, the maximum depth of penetration was 487 µm (Table S1, Supporting Information). The number of astrocytes in both cranial and caudal parts of the defect at the border with intact tissue was 20 cells on average (maximum density – 38 cells) (Figure 1d).

Histological analysis revealed infiltration of macrophages and newly emerged multiple clusters of glial cells, which were spread unevenly over the lesion on day 14 post‐SCI (Figure 1d). The scar tissue was characterized by a regular vascularization pattern, and the blood vessels returned to an ordinary histological organization. During the chronic stage, on day 30, multiple macrophages were still present and distributed evenly throughout the lesion in the spinal cord. We observed multiple newly formed blood vessels spread over the examined areas and reactive activation of astroglia in the peripheral portion of the lesion at the projection of ventral funiculi of spinal white matter (Figure 1d). By day 60, all animals had spinal cord lesions including large cystic cavities and a mature fibrous component. In some animals, racemose cavities were found cranial to the defect, along the spinal (central) canal, indicating impaired liquor dynamics (Figure 1d).

We next utilized LS‐MS/MS proteomics to elucidate global changes in proteostasis and signaling pathways, which are activated at the injury site, in our SCI model in comparison with compression impact. The absolute majority of proteins, upregulated in the damaged spinal cord of compression SCI, were also observed in the site of the injury and adjacent segment isolated from rats after cryoapplication (Figure 1e). Our data suggest that platelet degranulation, formation of fibrin clot, MAPK/MAPK2 signaling, regulation of IGF transport and uptake, and metallothioneins are similarly elevated during the acute phase of SCI in both models (Figure 1f). Importantly, MAPK signaling was previously reported as one of the major drivers of microglia‐mediated neuroinflammation during SCI.^[^ ^65^ ^]^ Observed elevation of the level of serine protease inhibitors (Serpins), C‐reactive protein (CRP), inter‐alpha‐trypsin inhibitor 4 (Itih4), kininogen 1 (Kng1), fibrinogen gamma and beta chains (Fgg/Fgb) is in line with recent studies, suggesting them as putative biomarkers of SCI in humans and rodents.^[^ ^66^, ^67^, ^68^, ^69^ ^]^ Upregulation of IGF‐related pathways and metallothioneins 1 and 2 (Mt1/Mt2) corresponds with its protective role in SCI pathogenesis.^[^ ^70^, ^71^, ^72^ ^]^ Western blotting analysis for SCI‐related markers revealed that the level of T‐box transcription factor 21 (Tbx21), GFAP, b‐arrestin‐2, and CD14 is elevated in the injured spinal cord isolated from rats with cryoapplication and compression SCI in comparison with intact animals.

### 3.2 SCI is Accompanied by Systemic Cytokine Storm in Rats and Humans

**Figure 2:**
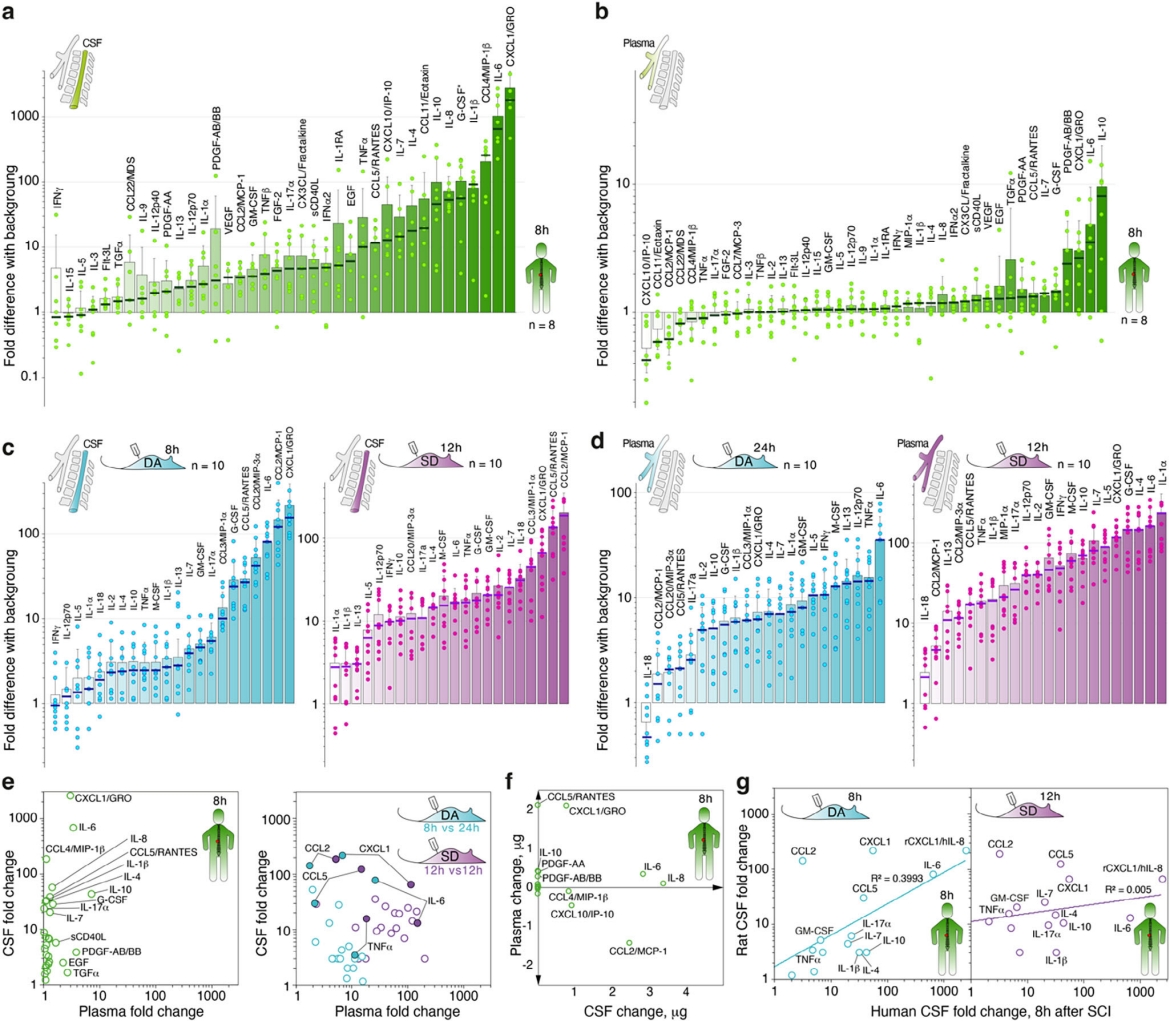
SCI dynamics in rats and humans during the acute phase is accompanied by systemic release of cytokines and chemokines. Increase of the level of cytokines and chemokines (fold change difference to background, 8–24 h after spinal cord injury as indicated) in CSF a–c) and plasma b–d) of humans subjected to surgery for intramedullary spinal cord tumors, SD and DA rats with experimental SCI. Bars represent average and standard deviation. Median is shown by a bold line. Dots represent individual values. e) Correlation of plasma and CSF cytokines and chemokines release in patients (green circles on left panel) or DA (violet) and SD (cyan) rats after SCI (right panel). f) Absolute change in quantity of cytokines and chemokines in plasma and CSF of patients. g) Correlation of CSF cytokines upregulation in humans and DA (left panel, cyan) or SD (right panel, violet) rats with SCI. As rodents lack IL‐8, rCXCL1/hIL‐8 denotes comparison of rat CXCL1 and human IL‐8. Abbreviations list: interleukin (IL), chemokine C‐C motif ligand (CCL), chemokine (C‐X‐C motif) ligand (CXCL), granulocyte colony‐stimulating factor (G‐CSF), tumor necrosis factor (TNF), interferon gamma (IFNγ), regulated on activation, normal T cell expressed and secreted (RANTES/CCL5), monocyte chemoattractant protein (MCP), macrophage inflammatory protein (MIP), platelet derived growth factors AA and AB/BB (PDGF‐AA/‐AB/BB), granulocyte‐macrophage colony‐stimulating factor (GM‐CSF), fibroblast growth factor 2 (FGF‐2), vascular endothelial growth factor (VEGF), epidermal growth factor (EGF), transforming growth factor alpha (TGFα), FMS‐like tyrosine kinase 3 ligand (Fit‐3L), interferon gamma‐induced protein 10 (IP‐10), fibroblast growth factor 2 (FGF‐2), macrophage colony‐stimulating factor (M‐CSF). Data on G‐CSF* values correspond to 12 h after surgery.

Current data on cytokine profiles during SCI are significantly varied due to different experimental animal models, time of sampling, and measurement techniques. We thus analyzed the release of cytokines and chemokines in plasma and cerebrospinal fluid (CSF) of DA and SD rats after SCI in comparison with human patients subjected to surgical treatment of intramedullary spinal cord tumors (**Figure** **2**a–d, Table 1). The choice of patients undergoing elective surgery instead of traumatic SCI was reasoned by several aspects.

Ependymomas represent the suitable nosology for this study as they are most commonly found in the cervical spine. In contrast to traumatic SCI, the surgical technique and approach employed in the treatment of ependymomas is relatively standardized, making this tumor type a reliable model. Indeed, removal of intramedullary spinal cord tumors is invariably accompanied by some degree of surgical trauma. Quantitative parameter – tumor size, which generally reflects the volume of surgical intervention, might be correlated with the amplitude of cytokine storm. Traumatic SCI is often accompanied by concomitant injury; thus, cytokines and chemokines released by various injured tissues would significantly disperse the isolated cytokine storm, which corresponds specifically to the damaged spinal cord. In case of traumatic SCI, neither synchronization of sampling time of plasma and CSF between patients nor background cytokine values are available. Finally, the installation of an epidural catheter to patients with traumatic SCI, especially during the acute phase, is potentially threatening for medical reasons.

Our data revealed that a massive cytokine storm in the central nervous system in humans during the acute phase, 8–24 h after SCI, is driven by neutrophil chemoattractant, chemokine (C‐X‐C motif) ligand 1 (CXCL1/GRO), which is upregulated three orders of magnitude above the basal level in the CSF. This chemokine burst is accompanied by 40 to 600‐fold increase in the CSF level of IL‐1β, IL‐6, IL‐8, IL‐10, macrophage inflammatory protein 1β (CCL4/MIP‐1β), and granulocyte colony‐stimulating factor (G‐CSF). Similarly, in rats with SCI, we observed a 10 to 200‐fold increase in the CSF concentration of CXCL1, IL‐6, MIPs 1α (CCL3) and 3α (CCL20), monocyte chemoattractant protein‐1 (CCL2/MCP‐1), CCL5/RANTES, and G‐CSF. As anticipated, cytokine and chemokine values were less spread in rats in comparison with humans due to different tumor sizes and possible individual deviations of immune status in patients. Cytokines and chemokines were mainly accumulated in CSF in humans and DA rats, whereas in SD their concentration following trauma was also relevant in the plasma (Figure 2e; Table S2, Supporting Information).

Absolute counting of distribution of cytokines and chemokines between plasma and CSF in humans with SCI revealed that CCL5, IL‐10 and platelet derived growth factors AA and AB/BB (PDGF‐AA/‐AB/BB) were released mainly into the plasma, whereas IL‐6, IL‐8, CCL2, CCL4 and interferon gamma‐induced protein 10 (CXCL10/IP‐10) were concentrated mainly in CSF (Figure 2f; Table S2, Supporting Information). Decrease of CCL2 and CXCL10 levels in plasma of humans with SCI, which is also clearly seen on Figure 2b, suggests migration of a significant portion of monocytes and macrophages from the periphery to the injury site. Inflammation burst in humans was ≈10 times more intense and correlated with those in DA and less with SD rats (Figure 2g; Table S2, Supporting Information).

**Figure 3:**
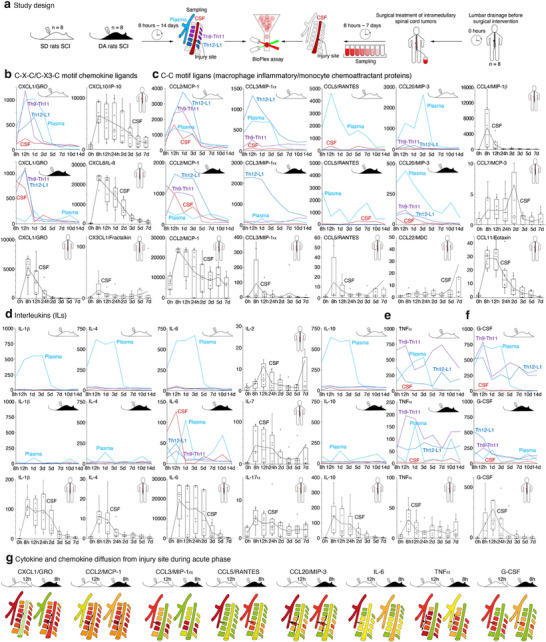
Dynamics of cytokine storm in humans, DA and SD rats after SCI. a) Study design and samples timeline. b–f) Cytokine and chemokine levels in plasma and CSF of humans subjected to surgical treatment of intramedullary spinal cord tumors in comparison with plasma (blue), CSF (red), injured (deep blue), and adjacent (violet) fragments of spinal cord of DA (black) and SD (white) rats after SCI. Bars represent median, interquartile range, and data spread interval. g) Schematic representation of cytokine and chemokine diffusion during the acute phase of SCI in DA and SD rats. For the cytokine and chemokine abbreviations list, please refer to Figure 2 legend.

We further analyzed the dynamics and the localization of systemic cytokine storm by measuring cytokine and chemokine levels in plasma and CSF of humans with SCI in comparison with plasma, CSF, injured and adjacent fragment of spinal cord of DA and SD rats after SCI (**Figure** **3**a; Tables S3 and S4, Supporting Information). The majority of analytes, except TNFα and negative regulator of ependymal cell proliferation – IL‐17α,^[^ ^73^ ^]^ peaked during the acute phase after SCI (0–48 h) and gradually decreased to background level by the end of the subacute phase (Figure 3b–g). Injured area in DA and SD rats exhibited a significantly increased concentration mainly of CXCL1, CCL2, CCL3, IL‐6, TNFα and G‐CSF. TNFα was readily detected in the nearby segment, confirming the observation of Springer et al., stating that upstream and downstream components of the caspase‐3 apoptotic pathway are activated after SCI in rats distant from the injury site.^[^ ^74^ ^]^ Importantly, the level of CX3CL1, CCL5, CCL22, IL‐2, and IL‐7 tended to increase in CSF of humans by day 7 after SCI, suggesting a recruitment of T cells into the CNS.

### 3.3 Amplitude of Cytokine Storm is Correlated with Size of Injured Area and Neurological Impairment in Humans

**Figure 4:**
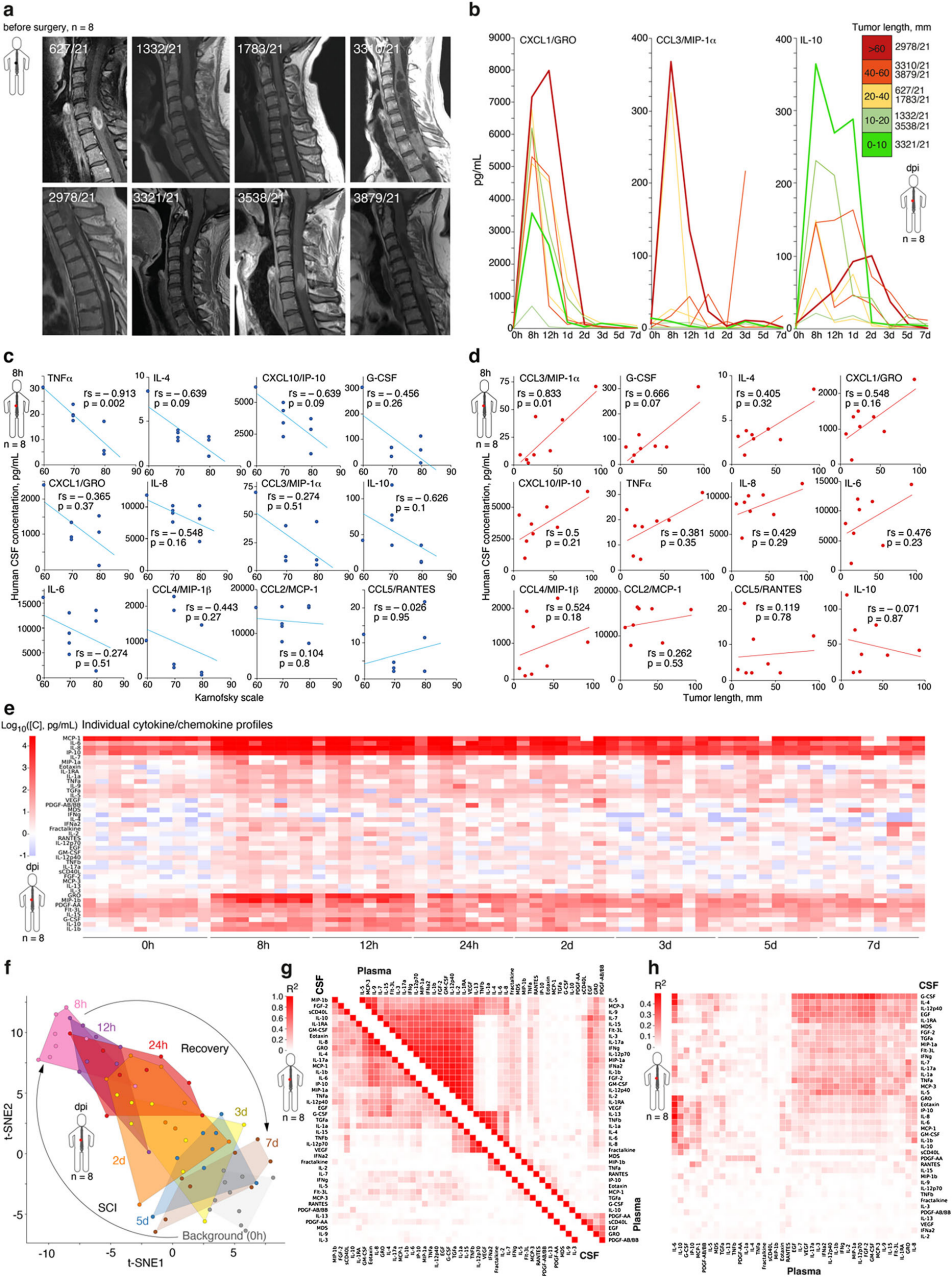
Amplitude of cytokine storm is correlated with tumor size and neurological impairment in humans a) MRI of patients with intramedullary spinal cord tumors before surgery. b) Individual changes of CXCL1, CCL3, and IL‐10 concentration in CSF of patients with intramedullary spinal cord tumors after surgical intervention. The insert shows the patient's code and tumor length in mm. Curves representing individual patients are colored according to the tumor length. c,d) Correlation of concentration of CSF cytokines and chemokines in humans after surgical treatment of intramedullary spinal cord tumors (8 h) during the acute SCI phase with Karnofsky scale at discharge (c) and tumor length (d). Spearman's rank correlation coefficient (r_s_) and two‐tailed p values are indicated. e,f) Patients’ individual cytokine and chemokine profiles (e) and t‐distributed stochastic neighbor embedding (t‐SNE) according to these data before surgery (0 h) and after SCI during the acute and sub‐acute phase (8 h – 7 days). g) Correlation of cytokines and chemokines upregulation during acute SCI phase in CSF or plasma. h) Cross‐correlation of plasma and CSF cytokines and chemokines during acute SCI phase. For the cytokine and chemokine abbreviations list, please refer to the Figure 2 legend.

We next aimed to match upregulation of cytokines and chemokines with tumor volume (**Figure** **4**a) and neurological outcomes (**Table** **2**).

**Table 2:** Demographic and clinical data of patients who underwent surgery due to an intramedullary spinal cord tumor.

Patient #	627/21	1332/21	1783/21	3310/21	2978/21	3321/21	3538/21	3879/21
IMSCT location ^a)^	C5‐C6	C3‐C4	C5‐C6	Th1‐Th2	C6‐Th3	C3	C5‐C6	C5‐Th1
Length according to FSU	1	1	1	1	3	1	1	2
Length, mm^b)^	26.0	15.9	24.0	56.8	94.2	84.0	18.1	43.2
Presence of a tumor cyst	Yes	yes	yes	yes	yes	no	no	yes
Sex	F	F	F	M	F	F	M	F
Age	30	48	28	32	57	54	34	60
Histology	Anaplastic ependymoma	Ependymoma	Anaplastic ependymoma	Ependymoma	Ependymoma	Ependymoma	Ependymoma	Ependymoma
Grade	3	2	3	2	2	2	2	2
ICD‐O code	9392/3	9391/3	9392/3	9391/3	9391/3	9391/3	9391/3	9391/3
Karnofsky scale								
Before surgery	90	90	90	70	70	90	80	70
At discharge	80	80	80	70	60	70	70	70

IMSCT – intramedullary spinal cord tumor; C – cervical spine; Th – Thoracic spine; FSU – functional spinal units; F – female; M – male; CSF – cerebrospinal fluid.

^a)^ The tumor location was defined in relation to the corresponding vertebrae;

^b)^ The length of the lesion was calculated by measuring the most cranial and caudal points of the tumor on the MR‐tomogram, with the greatest contrasting volume of the tumor.

Patient‐specific analysis of amplitude and duration of cytokine and chemokine persistence in CSF of humans after SCI revealed that proinflammatory chemokines CXCL1 and CCL3 tend to positively correlate with length of tumor, whereas anti‐inflammatory cytokine IL‐10 correlates negatively (Figure 4b). Further pairwise comparison of human CSF cytokine and chemokine burst relative to Karnofsky scale at hospital discharge (Figure 4c) and tumor length (Figure 4d) indicated that the level of TNFα, IL‐4 and CXCL10 negatively correlates with beneficial neurological outcome, while the level of CCL3 and G‐CSF correlates positively with tumor length. The IL‐10 was the unique cytokine, which tends to increase oppositely to the length of the tumor.

Analysis of patients’ individual cytokine and chemokine profiles revealed a very similar pattern of its upregulation in CSF after SCI (Figure 4e). Principal component analysis (PCA) and t‐distributed stochastic neighbor embedding (t‐SNE) revealed that the generalized vector of cytokine storm was equal in all patients (Figure 4f). These data suggest that cytokine and chemokine levels were significantly more homogeneous in terms of time post SCI rather than were patient‐specific. Correlation of cytokines and chemokines upregulation in CSF demonstrated two clusters, including (i) CXCL1/GRO, IL‐8, CCL11/Eotaxin, IL‐4, and (ii) lymphotoxin‐alpha (LTα/TNFβ), IL‐12p70, which were cooperatively elevated in patients after SCI (Figure 4g). Evaluation of plasma cytokines and chemokines (Figure 4g) revealed the existence of a massive cluster, containing more than 20 linked representatives (e.g., interleukins 1b, 1RA, 2, 3, 5, 7, 9, 12p40/70, 15, 17a, IFN γ, GM‐CSF, VEGF, CCL3/MIP‐1α and CCL7/MCP‐3). Meanwhile, only IL‐6 in plasma and G‐CSF in CSF tend to cross‐correlate with CSF and plasma cytokines and chemokines, respectively (Figure 4h). Thus, serum IL‐6 seems to be the most potent biomarker, reflecting the amplitude of SCI‐induced cytokine storm in CSF.

### 3.4 Infliximab and Mogamulizumab Reduce Inflammation in Injured Spinal Cord and Improve Locomotor Functions in SD Rats After SCI

**Figure 5:**
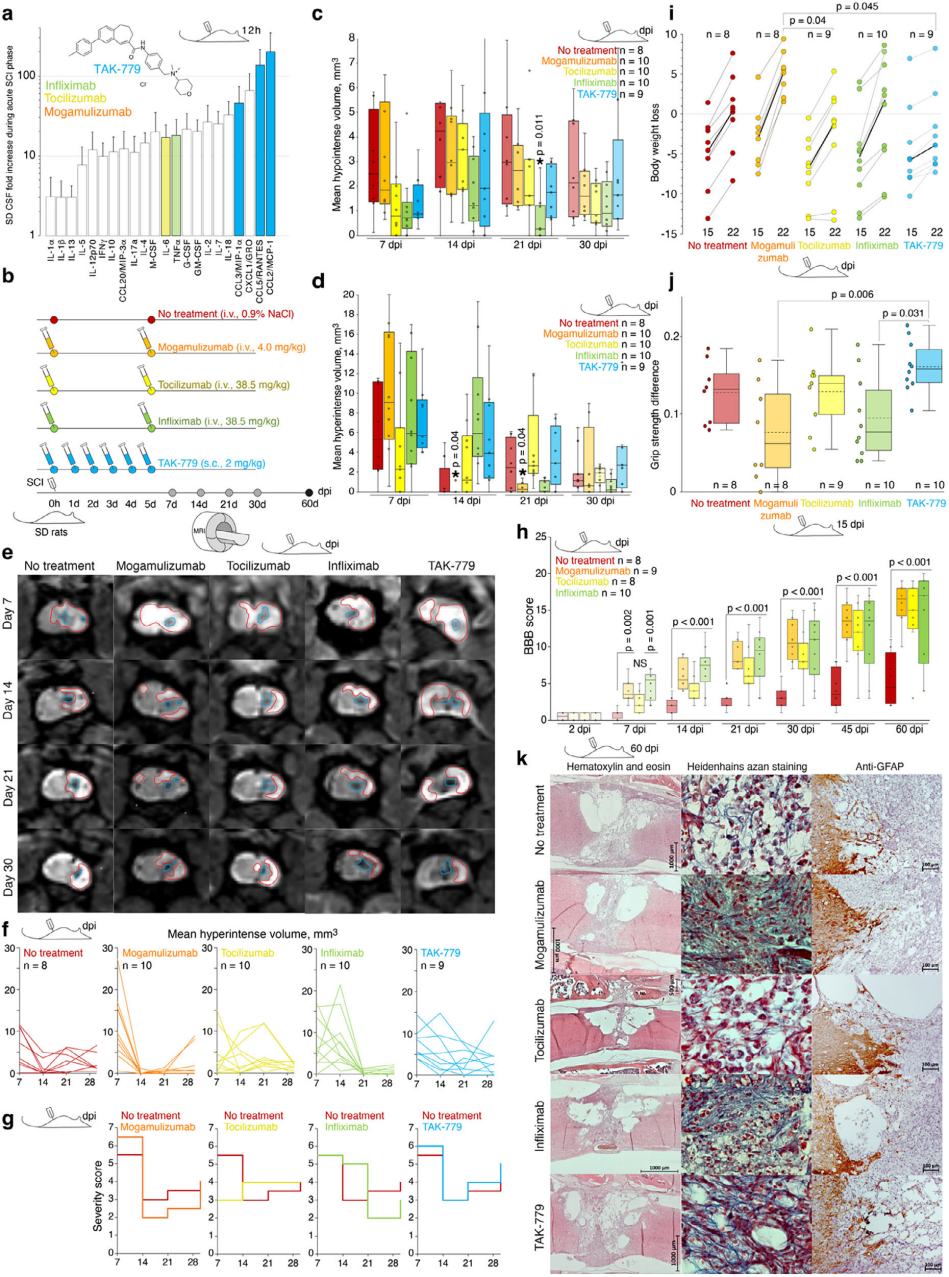
Infliximab and mogamulizumab reduce inflammation in the injured spinal cord and improve locomotor functions in SD rats after SCI. a) Increase of the level of cytokines and chemokines (fold change difference to background, 12 h after spinal cord injury) in CSF of SD rats after SCI. Colors indicate cytokines and chemokines targeted by respective therapeutics. Bars represent average and standard deviation. The chemical structure of TAK‐779 is shown. For the cytokine and chemokine individual values and abbreviations list, please refer to Figure 2. b) Scheme of administration of monoclonal antibodies and chemical compounds. Grey dots denote MRI tests, black dot – study termination. Dpi, days post injury. c,d) Mean area of hypointense (c) and hyperintense (d) lesions in the spinal cord of SD rats treated by mogamulizumab (orange), tocilizumab (yellow), infliximab (green), and TAK‐779 (blue) in comparison with untreated rats (red) with SCI. Bars represent median, interquartile range, and data spread. The asterisk denotes a statistically significant difference according to Tukey's honest significant difference (Tukey's HSD) test between treated and non‐treated groups. Respective p‐values are indicated. e) Representative spinal cord MRI of the injured segment of treated rats in comparison with non‐treated SD rats. Hyperintense and hypointense areas are shown by red and blue, respectively. f) Individual changes in the spinal cord hyperintense area in non‐treated SD rats and SD rats treated by indicated therapeutics. g) Mean MRI‐based semi‐quantitative severity score in groups of SD rats treated by indicated therapeutics in comparison with non‐treated SD rats. h–j) Mean Basso, Beattie, and Bresnahan (BBB) score (h), body weight loss 15‐22 days after SCI (i), and grip strength difference 15 days after SCI (j) values of non‐treated SD rats and SD rats treated by the indicated therapeutics. Statistically significant difference according to the Tukey's HSD test between the treated and control groups is indicated. Bars on (h) represent median, interquartile range, and data spread. Bold line on (i) indicates median shift. Bars on (j) represent median (bold), average (dotted), interquartile range, and standard deviation. k) Representative histoarchitectonics of the spinal cord injured site of SD rats treated by indicated therapeutics in comparison with non‐treated SD rats evaluated by Hematoxylin and eosin, Heidenhain's azan, and anti‐GFAP staining.

Comprehensive analysis of cytokine storm during SCI provided us with possible therapeutic targets, which may be affected by existing drugs already approved for medical usage or being currently involved in clinical trials. Assuming that CCL2, CCL3, and CCL5 were top upregulated chemokines in CSF of SD rats with SCI, we probed TAK‐779, a nonpeptide CCR5/CXCR3 antagonist.^[^ ^75^ ^]^ Together with this, we hypothesize that restriction of T cell infiltration, which was clearly observed during the subacute SCI period according to a specific chemokine burst in humans, may also be an effective therapeutic intervention. Thus, for such a purpose we recruited anti‐CCR4 antibody mogamulizumab.^[^ ^76^ ^]^ Taking into consideration previous reports.^[^ ^52^, ^53^, ^54^, ^55^ ^]^ and significantly elevated level of TNFα and IL‐6 in CSF of SD rats, we also tested two monoclonal antibodies, infliximab.^[^ ^77^ ^]^ and tocilizumab,^[^ ^78^ ^]^ respectively (**Figure** **5**a). All monoclonal antibodies under investigation were i.v. administrated twice, directly after SCI and 5 days after injury, in a dosage of 38.5 mg kg^−1^ (tocilizumab and infliximab) and 4 mg kg^−1^ (mogamulizumab) (Figure 5b). TAK‐779 in a dosage of 2 mg kg^−1^ was injected s.c. Directly after SCI and further daily for 5 days.

Injured animals were weekly monitored by magnetic resonance imaging (MRI) using specific non‐conventional sequences. In all groups postoperative soft tissue changes were visually observed at the level of the Th13 vertebra to the right of the midline in T1 and T2 modalities, and in the corresponding area of the spinal cord – an either rounded or longitudinally extending in the cranio‐caudal direction hypointense area up to 3.5 mm in length (corresponding to the length of the Th13 vertebra) and 0.5‐1.5 mm in diameter. Clear, irregular contours were visually connected with the adjacent spinal cord sheaths from the laterodorsal side and with the adjacent large vessels of the spinal cord (*a. spinalis dorsalis dextra, a. et v.spinalis ventralis*). The specified area presumably corresponds to acute consequences of the injury, including hematoma and later developing necrosis. Homogeneous dynamical evolution of the structure of this area was observed between the experimental groups, which reached its maximum volume by day 14 after the SCI. This was characterized by a shape close to a longitudinally oriented ellipsoid or “hourglass”. Further, the area gradually decreased in volume, taking the shape of a “crack” or “canyon” with a characteristic sinuous, branching course, presumably as a result of glial scar organization.

In the T2 mode, 7 days after the trauma, an extensive diffuse hyperintense area without clear contours was observed in the injury site, likely representing posttraumatic edema. The latter regressed almost completely in 30 days. At the same time, the volume of foci with hyperintense signal reached its maximum by 21 days after the trauma with preservation by day 60 due to the formation of rounded, highly hyperintense structures with clear contours located in close proximity to the central canal of the spinal cord, presumably posttraumatic cysts, as well as hyperintense areas indicating central canal expansion of “posttraumatic syringomyelia‐like” type.

Analysis of the volume of hypointense (Figure 5c) lesions revealed a statistically significant decrease in the group treated by infliximab up to three weeks following SCI. Hyperintense volume in the injured spinal cord was significantly reduced in groups treated by mogamulizumab on 14–21 days (Figure 5d). Even more importantly, to monitor each animal dynamically and compare the level of initial neurological dysfunction with treatment progress as we, clinicians, do in case of our patients. Analysis of individual MRI data (Figure 5e–f) and median severity score (Figure 5g) confirmed the therapeutic effect of mogamulizumab in a period from 2 to 3 weeks after SCI. Even though locomotor activity evaluation according to Basso, Beattie and Bresnahan (BBB) score revealed that all groups treated by monoclonal antibodies, but not TAK‐779, recovered significantly faster in comparison with non‐treated animals (Figure 5h; Figure S1, Supporting Information), evaluation of body weight loss (Figure 5i) and grip strength difference (Figure 5j) caused by trauma again demonstrated beneficial effect of mogamulizumab and infliximab over TAK‐779 and tocilizumab on 22 and 15 day post injury.

### 3.5 Administration of Mogamulizumab Minimizes Defect Area and Cyst Formation in SD Rats After SCI

Pathohistological evaluation of the therapeutic potential of the selected agents was limited exclusively to the 60th day, since the key events in the course of natural formation and maturation of glial scar tissue after SCI in rats were completed by the above‐mentioned period. By the 60th day of observation, in all groups, the defects had an “hourglass” appearance, did not extend beyond the access vertebra (Th13), and were transmural since they involved the entire dorso‐ventral complex of the spinal cord (Figure 5k; Table S5, Supporting Information). The glial scar in SD rats after SCI, treated by mogamulizumab, was the most compact and significantly differed from non‐treated animals.

The number of macrophages in the spinal cord glial scar of SD rats treated with tocilizumab, infliximab, and mogamulizumab by the end of 60 days exceeded those in animals without treatment (Table S6, Supporting Information). Though the increased number of macrophages differently affected the configuration, density, and distribution of collagen fibers in the scar tissue. Low content of collagen fibers, practically 2 times less than in non‐treated animals, was observed in the case of tocilizumab administration, while injection of infliximab and mogamulizumab promoted a denser fibrous framework of the glial scar (Table S6, Supporting Information). Increased presence of the fibrous component in the groups treated by infliximab and mogamulizumab appeared to be associated, first of all, with the central part of the defect in the dorso‐ventral direction. It took the form of a denser “core” with a rougher scar structure and thickening of collagen fiber bundles. At the same time, the observed relative volume of vessels in the spinal cord defect 2 months after the SCI in the case of administration of monoclonal antibodies did not differ from the non‐treated rats (Table S6, Supporting Information).

The spinal cord defect in SD rats treated by tocilizumab 60 days post SCI was always heterogeneous in structure, by 30‐75% consisted of huge cyst‐like cavities up to 1.0 x 1.7 mm in the projection of dorsal tubules of white matter and gray matter on its border with intact tissue (Figure 5k). Sometimes, small‐size cystic cavities were also located in the projection of ventral tubules of white matter. In case of administration of infliximab, we also detected cystic cavities, but the histoarchitectonics of the glial scar was more dense and homogeneous. Statistically significant difference in total area of cystic cavities in comparison with non‐treated animals was observed among the SD rats with SCI treated with mogamulizumab (Table S7, Supporting Information). The sizes of cystic cavities were directly proportional to the linear sizes of the formed spinal cord defects (Tables S5 and S7, Supporting Information), and, in the case of tocilizumab, inversely proportional to the relative volume of the fibrous component (Tables S6 and S7, Supporting Information).

The average density of GFAP‐positive cells at the border with intact tissue and their average penetration into the depth of the defect did not show any significant differences among all test groups. However, the maximum penetration depth of GFAP‐positive cells closely correlated with the glial scar density. Thus, the maximum indirect manifestations of axonal regeneration in terms of the depth of penetration of GFAP‐positive cells into the glial scar structure were observed in SD rats treated with tocilizumab (Table S8, Supporting Information).

### 3.6 TAK‐779 and MK‐7123 Reduce Inflammation in Injured Spinal Cord During Acute Phase in DA Rats After SCI

**Figure 6:**
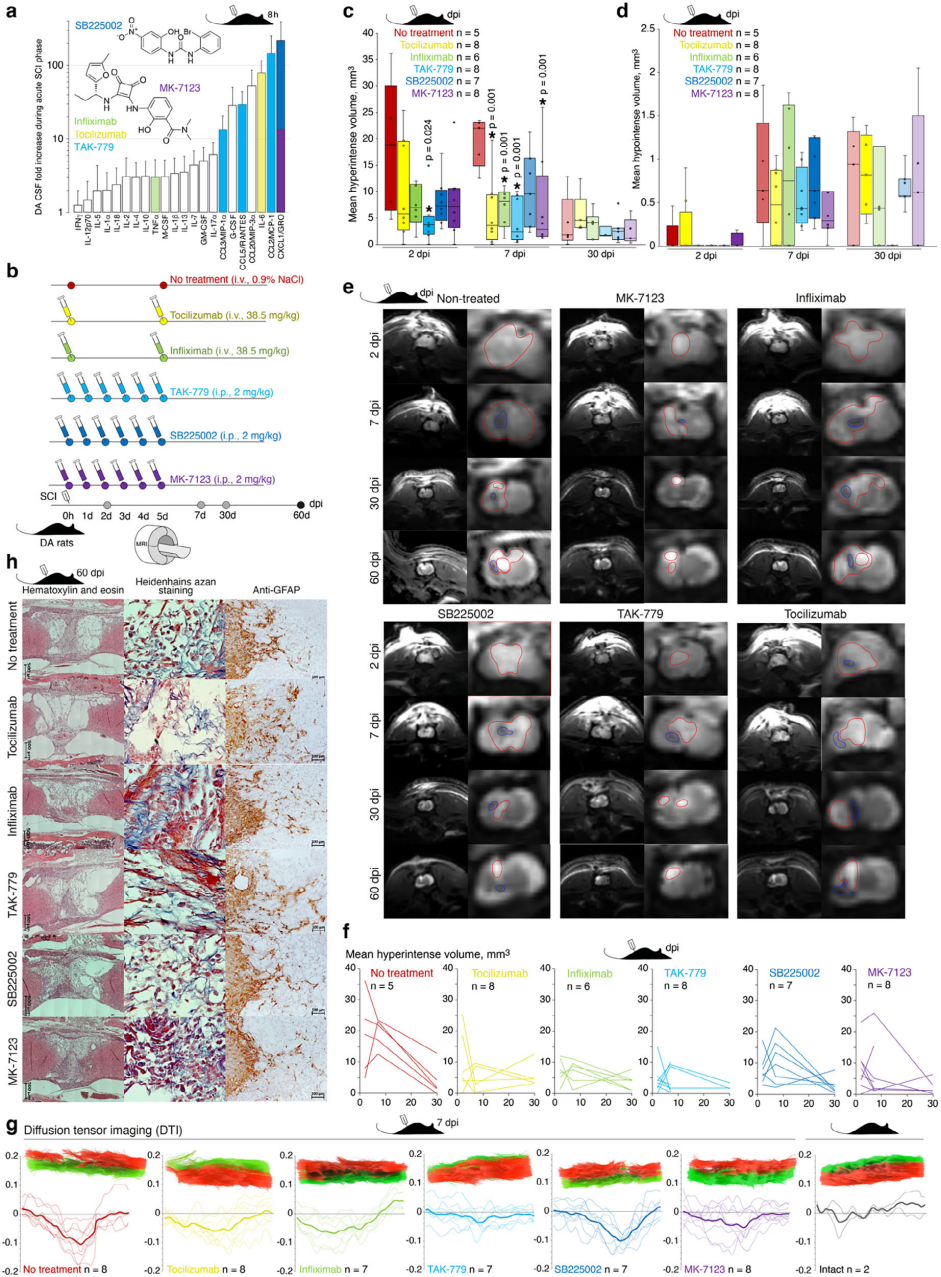
TAK‐779 and MK‐7123 reduce inflammation in injured spinal cord during acute phase in DA rats after SCI a) Increase of level of cytokines and chemokines (fold change difference to background, 8 h after spinal cord injury) in CSF of DA rats after SCI. Colors indicate cytokines and chemokines targeted by respective therapeutics. Bars represent average and standard deviation. Chemical structures of MK‐7123 and SB225002 are shown. For cytokine and chemokine individual values and abbreviations list please refer to Figure 2. b) Scheme of administration of monoclonal antibodies and chemical compounds. Grey dots denote MRI tests, black dot – study termination. Dpi, days post injury. c,d) Mean area of hyperintense (c) and hypointense (d) lesions in the spinal cord of DA rats treated by tocilizumab (yellow), infliximab (green), TAK‐779 (blue), SB225002 (deep blue), and MK‐7123 (violet) in comparison with untreated rats (red) with SCI. Bars represent median, interquartile range, and data spread. The asterisk denotes a statistically significant difference according to Tukey's HSD between treated and non‐treated groups. Respective p‐values are indicated. e) Representative spinal cord MRI of the injured segment of treated DA rats in comparison with non‐treated animals. Hyperintense and hypointense areas are shown by red and blue, respectively. f) Individual changes in the spinal cord hyperintense area in non‐treated DA rats and DA rats treated and by indicated therapeutics. g) Diffusion tensor imaging (DTI) of the injured area of treated and non‐treated DA rats on day 7 after SCI. Individual traces and the average curve for each group are shown by thin and thick lines, respectively. Representative reconstruction of fiber tractography is shown on top. h) Representative histoarchitectonics of the spinal cord injured site of DA rats treated by indicated therapeutics in comparison with non‐treated DA rats evaluated by Hematoxylin and eosin, Heidenhain's azan, and anti‐GFAP staining.

Profiling of CSF cytokine and chemokine upregulation during SCI modelling in DA rats revealed the leading role of CXCL1 and IL‐6, which reflects those in humans (Figure 2a–c). Therefore, for treatment of SCI in DA rats, in addition to TAK‐779 and infliximab, we chose tocilizumab and two low molecular weight antagonists of CXCR1/CXCR2 receptors, SB225002.^[^ ^79^ ^]^ and MK‐7123.^[^ ^80^ ^]^ (SCH 527123 or navarixin) (**Figure** **6**a). Injection of monoclonal antibodies was accomplished in a regime similar to SD rats. Lack of therapeutic effect of TAK‐779 in SD rats forced us to change the administration route of chemical compounds from s.c to i.p. in order to increase its bioavailability and prolong the release to the bloodstream (Figure 6b). Infliximab, TAK‐779, and MK‐7123 decrease the volume of hyperintense lesions on day 7 after SCI, whereas TAK‐779 also significantly reduces inflammation during the acute phase of SCI (Figure 6c). No statistically significant difference between treated and untreated rats was observed in the volume of hypointense areas (Figure 6d). We also compared the effect of chemokine receptor antagonists with dexamethasone medication in the same dosage 2 mg kg^−1^ (2.5 mg per DA rat in total) as previous studies suggest its toxicity in case of 4 mg week^−1^ administration.^[^ ^81^ ^]^ Similar to the report by Kwiecien et al,^[^ ^82^ ^]^ our data demonstrate that administration of dexamethasone to injured animals had no beneficial effect on the inflammation process (Figure S2, Supporting Information).

Rat‐specific analysis (Figure 6e,f) also suggested that TAK‐779 and MK‐7123 are the most effective in diminishing of inflammation during first week after SCI. In order to analyze this in more details, we performed diffusion tensor imaging (DTI) of injured area of rats on day 7 after SCI. Difference in functional anisotropy between right (injured) and left (intact) spinal cord lateral halves confirmed our previous conclusions and revealed that SCI treatment by MK‐7123 and in particular TAK‐779 preserve native structure of spinal cord (Figure 6g; Video S2–S8, Supporting Information).

### 3.7 Administration of Infliximab and TAK‐779 Increase Depth of Penetration of GFAP‐Positive Cells into the Defect Area in DA Rats After SCI

Similar to SD rats, we histologically analyzed the final outcomes of the applied therapy in DA rats (Figure 6h). By the 60th day of observation, the linear characteristics of the glial scar of the spinal cord in DA rats from different groups were largely variable and not statistically different (Table S9, Supporting Information). The largest size of glial scars, 4.3 ± 0.6 mm^2^ with an average size of cranio‐caudal defect of 4.3 ± 0.5 mm, was observed in the case of tocilizumab administration. The spinal cord defects in DA rats were always heterogeneous in structure, and at least 20% contained cyst‐like cavities of variable size (Table S10, Supporting Information). The largest cysts were located in the cranial part of the defect in the projection of the dorsal tubules of the white matter and in the projection of the gray matter on its border with the intact tissue (Figure 6h). Small cystic cavities were located in the projection of the ventral tubules of the white matter. Normally, the sizes of cystic cavities were directly proportional to the linear sizes of the spinal cord defects (Tables S9 and S10, Supporting Information).

In the injured area of rats receiving saline, the average number of macrophages was 23 ± 2 cells. Administration of monoclonal antibodies, but not chemical compounds, led to a significant elevation of macrophage presence (Table S11, Supporting Information). Treatment of DA rats by infliximab and tocilizumab in comparison with non‐treated animals resulted in higher (0.27 ± 0.05 mm^3^/mm^3^) and lower (0.14 ± 0.03 mm^3^/mm^3^) content of collagen fibers in the structure of glial scars of the spinal cord (Table S11, Supporting Information). Administration of low molecular weight substances did not cause significant modification of the fibrous component of the glial scars, with the average value of the relative volume of collagen fibers being slightly lower than that in the untreated group (Table S11, Supporting Information). Relative volume of vessels in the structure of the spinal cord defect did not differ between treated and non‐treated animals (Table S11, Supporting Information). In the aspect of axonal regeneration criteria, all tested therapeutic agents in comparison with non‐treated animals, demonstrated a positive trend in terms of average depth of astroglia penetration into the defect structure. Administration of infliximab and, more particularly, TAK‐779 to DA rats after SCI resulted in most pronounced penetration of GFAP‐positive cells into the glial scar structure (Table S12, Supporting Information).

## 4 Discussion

The removal of intramedullary spinal cord tumors causes surgical trauma by a different extent, which is contingent upon the anatomical location, size, histological classification, and growth pattern of the tumor, invasive or detached.^[^ ^59^ ^]^ The most common spinal cord tumors are ependymomas, hemangioblastomas, and astrocytomas. Furthermore, astrocytomas frequently exhibit an infiltrative growth pattern, while haemangioblastomas may display intra‐extramedullary spread and unique challenges in surgical removal. The growth of ependymomas most often originates from the lining of the central canal of the spinal cord.^[^ ^83^ ^]^ In general, ependymomas are localized in the cervical spine and are benign tumors (the majority are grade 2). They are separated from the neural tissue and routinely subjected to total removal.^[^ ^58^ ^]^ In order to approach these tumors during surgical treatment, a myelotomy is performed, then the tumor is isolated cranially and caudally and removed, if possible, as a single block. We therefore chose ependymomas as a generalized model of molecular and immunological processes occurring exactly in the damaged spinal cord to elucidate cytokine and chemokine pathways, which may be purposefully blocked.

To evaluate the factors affecting cytokine profile in humans, we performed correlation analysis between tumor size and the functional status of patients with the concentration of cytokines in plasma and CSF. Here we show that, similar to serum zinc concentration,^[^ ^84^ ^]^ level of TNFα, IL‐4, and CXCL10 in CSF may be used as a predictive value of SCI outcomes in humans. Additionally, the cytokine profile was compared with the results of rodents with SCI. Certainly, the limitations of this approach include different methods of modeling spinal injury for humans and animals, as well as a significant difference in the volume of injury inflicted to animals by cryoapplication and precision surgery of spinal cord tumors. However, these models are based on the unity of neural tissue damage: primary, mechanical or thermal, and secondary injury, defined by pathologic changes at the cellular and molecular level.^[^ ^85^ ^]^ This approach is pioneering and allows us to overcome the limitations associated with extrapolating data from laboratory animals to humans. Importantly, our data suggest that, at least on the level of experimental SCI, compression injury of the spinal cord is very similar to utilized here cryoinjury model in terms of activated molecular pathways. Indeed, comparison of our SCI model with previously published reports describing contusion SCI^[^ ^86^, ^87^, ^88^ ^]^ reviled similar histoarchitectonics of glial scar and BBB score with 2–3 weeks delayed recovery of our animals (Figure 1d–h).

Recently, Mazzitelli et al. showed that the level of CCL2/MCP‐1 is significantly elevated during mechanical SCI in mice, while CXCL1/GRO, CCL3/MIP‐1α, IL‐6, and G‐CSF tend to increase.^[^ ^89^ ^]^ Authors further state that CSF‐derived signals contribute activation and recruitment of myeloid cells from bone marrow. These data correlate with our results, suggesting that cytokine release during SCI in mammals has a universal pattern regardless of species and traumatic mechanism and is not gender‐specific. Indeed, in our study not all cytokines were perfectly matched between humans and rodents. However, the level of major ones, namely CXCL1/IL‐8 and CCL2‐5, targeted by selected substances, was in a similar fashion dramatically upregulated (Figure 2d).

Modification of the histoarchitectonics of the glial scar in the spinal cord after SCI for better or worse outcome from the point of view of axonal regeneration is a debatable issue for several reasons. First, glial scar tissue should contain an optimal volume of collagen fibers, as it should be “loose” enough for axon growth. On the other hand, such tissue will be prone to an avalanche‐like increase of the bulky cystic cavities, which, in turn, will create an insurmountable barrier to axonal regeneration. In contrast, a denser defect with a high collagen fiber content will minimize the size of the cyst‐like cavities but will create a difficult permeable barrier to axonal growth. Second, a very important criterion for axonal regeneration is the level of tissue trophicity. In this regard, it is essential that, as the glial scar matures, there would be no obliteration of already formed vessels. Such a process would increase the relative volume of collagen fibers, which, in turn, would again hinder axonal regeneration. Third, the macrophages, monocytes, and neutrophils are undoubtedly the key players in the formation of glial scar tissue. Consequently, the count of these cells in the defect should be optimal both for reasonable attraction of fibroblasts and for the occurrence of neoangiogenesis.^[^ ^90^ ^]^

Our histological studies have convincingly proved that the histoarchitectonics of the glial scar as a result of SCI is amenable to significant therapeutic modification by suppressing the early waves of cytokine posttraumatic storm, mainly in terms of the content of collagen fibers and the extent of cyst formation. Therapeutic monoclonal antibodies promoted long‐term increased presence of macrophages in the structure of the glial scar, especially in the case of tocilizumab and mogamulizumab. At the same time, they affected the structure of the defect differently. Thus, in the case of tocilizumab, the glial scar in terms of collagen fibers synthesis appeared to be the most “loose” with consequent formation of the more relevant size of cystic cavities with minimal specific volume of fibrous component. It is evident that the increase in the size of post‐traumatic cystic cavities in the case of tocilizumab administration will undoubtedly hinder axonal regeneration. On the contrary, administration of infliximab and mogamulizumab leads to a dense glial scar with a minimum size of cystic cavities and high relative volume of collagen fibers. Nonetheless, this is also true for the situation when minimization of cyst size is achieved at the expense of excessive glial scar density. Future studies should demonstrate if application of neuroepithelial‐like stem cells (NESCs).^[^ ^91^ ^]^ together with probed therapeutics, may optimize density and cyst volume for axonal regrowth.

Usage of therapeutic antibodies basically does not affect the dynamics of neoangiogenesis in the glial scar of the spinal cord on long‐term follow‐up. This seems to be important in terms of the new role of microvessels in engulfing myelin debris and fibrosis after neural injury.^[^ ^92^ ^]^ As for low‐molecular‐weight substances, administration of TAK‐779 proved to be close to the effect of tocilizumab, while SB225002 and MK‐7123 did not significantly modify the glial scar of the spinal cord in DA rats. Our data indicate that the administration of the majority of probed therapeutics practically does not affect the density of astrocytes and the average depth of their penetration into the defect structure; however, treatment by infliximab and TAK‐779 promotes the maximum deep penetration of astroglia in conditions of low content of collagen fibers.

The MRI monitoring revealed that following SCI treatment by infliximab and mogamulizumab, the volume of posttraumatic hyperintense area in the sub‐acute period is significantly diminished. In the case of administration of mogamulizumab, posttraumatic edema is significantly pronounced at one week post SCI, but from the 14th day it almost completely resolves, and the development of posttraumatic cysts was delayed from 30–40 days to up to two months after trauma. Importantly, mogamulizumab, which is approved for two types of cutaneous T‐cell lymphoma treatment,^[^ ^93^ ^]^ is also considering for the therapy of T‐cell leukemia virus type‐1 (HTLV‐1)‐associated myelopathy/tropical spastic paraparesis.^[^ ^94^ ^]^ During this disease, HTLV‐1‐infected CD4 T cells penetrate through blood blood‐brain barrier to the spinal cord and stimulate the release of CXCL10 by astrocytes, which recruits more CXCR3‐positive lymphocytes into CNS.^[^ ^95^ ^]^ Among clinical outcomes of mogamulizumab administration in patients with HTLV‐1‐associated myelopathy, Sato et al. showed decreased levels of CXCL10,^[^ ^96^, ^97^ ^]^ which is significantly elevated during SCI as well, and negatively correlates with the Karnofsky scale according to our data. Interestingly, the timing of the therapeutic effect of mogamulizumab covers an interval of 14–21 days after SCI. This observation can be explained by a decrease of lymphocyte infiltration, starting one week after SCI, as a result of the mogamulizumab administration.

Inflammation during the acute and first week of sub‐acute phases of SCI was mostly effectively suppressed by TAK‐779 and MK‐7123. These findings are in line with the direct linkage of CCR5 and CXCR1/CXCR2 with the MAPK signaling pathway,^[^ ^98^, ^99^ ^]^ which is highly upregulated in the injured spinal cord according to our data. Importantly, genes coding for CXCR2, CCR5, and its ligands CCL2 and CCL3 are overexpressed in border‐forming wound repair astrocytes up to two months after SCI.^[^ ^100^ ^]^ Therefore, administration of antagonists of chemokine receptors should be limited to the first days after SCI in order to neutralize the primary pathogenic chemokine burst and not to interfere with astrocytes' regenerative function. MK‐7123 was recently tested in clinical trials for solid tumors therapy.^[^ ^101^ ^]^ and earlier showed efficacy in treatment of severe asthma.^[^ ^102^ ^]^ and chronic obstructive pulmonary disease.^[^ ^103^ ^]^ Despite discontinuations with higher doses of MK‐7123 as reported by a decrease in absolute neutrophil count in the group that received 50 mg of MK‐7123 daily for 6 months reported by Rennard et al.,^[^ ^103^ ^]^ we suggest that a short course (1 week or even less) of MK‐7123 would be safe and beneficial for SCI treatment.

The main message of our study is that cytokines and chemokines starting minutes following trauma and lasting several weeks significantly affects the pathogenesis of SCI. Our data report several time‐resolved waves of cerebral cytokine storm, thus ongoing and future clinical trials involving anti‐inflammatory drugs should more carefully select the time window for the therapeutic intervention.^[^ ^32^ ^]^ Despite we still observe the effect of infliximab and tocilizumab on SCI course in terms of histoarchitectonics of glial scar, more accurate analysis, including precise non‐invasive instrumental methods such as MRI‐DTI on the level of individual animals, suggests that its effect is not unambiguous. Indeed, our study has a number of limitations, i.e., a mechanistically different methodology of spinal cord injury in the analyzed species; possible alterations in molecular pathways involved in SCI pathology in mammals, especially of traumatic etiology; a confined human cohort and the non‐obviousness of administration of cytokine and chemokine inhibitors to patients with massive concomitant injury. Future studies involving biobank/sample‐sparing protocols should elucidate if the suggested therapy may be safe and beneficial for traumatic human SCI. Concluding, the demonstrated universality of cytokine storm during SCI in humans and rats supports the feasibility and motivates clinical evaluation of the reported approach in terms of experimental therapy, first, indeed in rationally chosen SCI cohorts.

Evident outcomes of our study state that time‐related combined therapy by anti‐chemokine drugs, including the anti‐CXCR1/2 or ‐CCR5 antagonists and mogamulizumab, might ameliorate the evolution of SCI sequelae during the acute, sub‐acute, and chronic phases, respectively. As we showed that all these drugs act individually, treatment may be started immediately after SCI by chemokine inhibitors in terms of emergency medicine or after one or even two weeks by immunoglobulins inpatiently if the person is out of medical assistance at the time of injury. Indeed, the described approach is not limited to traumatic SCI and can also potentially be translated into the clinical practice of rehabilitation after elective surgery.

## Conflict of Interest

The authors declare no conflict of interest.

## Author Contributions

A.A.B., G.B.T. and A.S.C. contributed equally to this work. G.B.T., N.A.K., D.Y.U., F.A.M., A.S., A.A.B., A.G.G., and A.N.K. conceived the study and designed the protocol. A.N.M., A.S.C., and D.A.B. performed the animal surgeries. M.V.R. was responsible for MRI and CT studies. V.A.K. performed histological analysis. V.A.P. accomplished an assessment of the locomotor activity. R.H.Z., D.A.O., and A.S.E. performed mass spectrometry studies. A.A.B. and A.A.K. designed and performed cytokine profiling. D.S.A. and Y.M.P. collected SCF samples and prepared patients’ medical histories. A.A.B. designed and performed all the data analyses. G.B.T., A.S.C., Y.M.P., A.S.E., D.S.A., I.N.P., N.A.K., D.Y.U. and A.N.K. advised on the data analyses. A.A.B. wrote the manuscript with input from A.S.C., A.N.M., M.V.R., V.A.K., Y.M.P., A.S., D.S.A., A.G.G. and A.N.K.

## Supporting information



Supporting Information

Supplemental Figure 1

Supplemental Figure 2

Supplemental Table 2

Supplemental Table 3

Supplemental Table 4

Supplemental Video 1

Supplemental Video 2

Supplemental Video 3

Supplemental Video 4

Supplemental Video 5

Supplemental Video 6

Supplemental Video 7

Supplemental Video 8

## Data Availability

All data relevant to the study are included in the article or uploaded as supplementary information. Raw LC‐MS/MS data are deposited to the ProteomeXchange Consortium via the PRIDE partner repository with the dataset identifier PXD067635.
