# Supplementary material for: Repurposing of Chemokine Antagonists for Combined Phase‐Resolved Spinal Cord Injury Treatment
Source: Adv Sci (Weinh). 2025 Oct 28;13(1):e16569. doi: 10.1002/advs.202516569 (PMC12767007; doi:10.1002/advs.202516569)
Supplement: Supplementary file 1 — Supporting Information [file ADVS-13-e16569-s011.docx]

**Repurposing of chemokine antagonists for combined phase-resolved spinal cord injury treatment**

Alexey A. Belogurov Jr.^1,2,*,**^, Georgii B. Telegin^3,**^, Aleksandr S. Chernov^1,3,**^, Anna A. Kudriaeva^1^, Alexey N. Minakov^1,3^, Maksim V. Rodionov^4^, Vitaly A. Kazakov^3^, Viktor A. Palikov^3^, Yuri M. Poluektov^5^, Rustam H. Ziganshin^1^, Daria A. Orlova^1^, Alena S. Evpak^1^, Daniil A. Barsuk^3^, Fedor A. Mesheryakov^1,3^, Aldo Spallone^1,6^, Dmitry S. Asyutin^5^, Igor N. Pronin^5^, Nikolay A. Konovalov^5^, Dmitri Yu. Usachev^5^, Alexander G. Gabibov^1,*^, Alexander N. Konovalov^5,*^

^1^Shemyakin and Ovchinnikov Institute of Bioorganic Chemistry, Russian Academy of Sciences, Moscow, 117997, Russia;

^2^Department of Biological Chemistry, Russian University of Medicine, Ministry of Health of Russian Federation, Moscow, 127473, Russia

^3^Branch of Shemyakin and Ovchinnikov Institute of Bioorganic Chemistry, Russian Academy of Sciences, 142290 Pushchino, Russia

^4^Medical Radiological Research Center (MRRC) named after A.F. Tsyb, Branch of the National Medical Radiological Research Center, Ministry of Health of Russian Federation, 249031 Moscow, Russia

^5^N.N. Burdenko National Scientific and Practical Center for Neurosurgery, Ministry of Health of Russian Federation, 125047 Moscow, Russia

^6^Department of Clinical Neurosciences, NCL-Neuromed Institute of Neurosciences, Rome, Italy.

*These authors contribute equally

**Correspondence: [akonovalov@nsi.ru](mailto:akonovalov@nsi.ru), [gabibov@ibch.ru](mailto:gabibov@ibch.ru), [belogurov@ibch.ru](mailto:belogurov@ibch.ru)

Supplementary materials list

Supplementary Figures S1-S2

Supplementary Tables S1-S12

Supplementary Videos S1-S8

**Supplementary Figure 1, related to Figure 5.** Receiver operating characteristic area under the curve (AUC ROC) of BBB scores of treated and non-treated rats as indicated 60 days post injury.

**Supplementary Figure 2, related to Figure 6.** Mean area of hypointense and hyperintense lesions in spinal cord of rats treated by dexamethasone (grey) and untreated rats (red) with SCI. Bars represent median, interquartile range and data spread.

**Supplementary Table 1, related to Figure 1.** Count of GFAP-positive cells in a unit area of 500 x 500 μm on the border of the defect with intact tissue, average and maximum depth of its penetration into the structure of the glial scar of the spinal cord of SD rats at different time intervals after SCI

**Supplementary Table 2, related to Figure 2.** Increase of cytokines and chemokines level in plasma and CSF of humans and rats during acute phase of SCI.

**Supplementary Table 3, related to Figure 3.** Dynamics of cytokines and chemokines release in plasma, CSF, injured and adjacent fragment of spinal cord of DA and SD rats after SCI.

**Supplementary Table 4, related to Figure 3.** Dynamics of cytokines and chemokines release in plasma and CSF of humans subjected to surgical treatment of intramedullary spinal cord tumors.

**Supplementary Table 5, related to Figure 5.** Linear dimensions of the spinal cord defect (defect area, cranio-caudal and dorso-ventral dimensions) in SD rats treated by tocilizumab, infliximab and mogamulizumab 60 days post SCI in comparison with non-treated animals.

**Supplementary Table 6, related to Figure 5.** Number of macrophages, relative volume of collagen fibers and vessels in the structure of the glial scar in the spinal cord of SD rats treated by tocilizumab, infliximab and mogamulizumab 60 days post SCI in comparison with non-treated animals. The asterisk denotes a statistically significant difference between treated and non-treated groups.

**Supplementary Table 7, related to Figure 5.** Size of cystic cavities in the structure of the glial scar in the spinal cord of SD rats treated by tocilizumab, infliximab and mogamulizumab 60 days post SCI in comparison with non-treated animals. The asterisk denotes a statistically significant difference between treated and non-treated groups. The asterisk denotes a statistically significant difference between treated and non-treated groups.

**Supplementary Table 8, related to Figure 5.** Count of GFAP-positive cells in a unit area of 500 x 500 μm on the border of the defect with intact tissue, average and maximum depth of its penetration into the structure of the glial scar of the spinal cord of SD rats treated by tocilizumab, infliximab and mogamulizumab 60 days post SCI in comparison with non-treated animals.

**Supplementary Table 9, related to Figure 6.** Linear dimensions of the spinal cord defect (defect area, cranio-caudal and dorso-ventral dimensions) in DA rats treated by tocilizumab, infliximab, SB225002, MK-7123 and ТАК-779 60 days post SCI in comparison with non-treated animals.

**Supplementary Table 10, related to Figure 6.** Size of cystic cavities in the structure of the glial scar in the spinal cord of DA rats treated by tocilizumab, infliximab, SB225002, MK-7123 and ТАК-779 60 days post SCI in comparison with non-treated animals. The asterisk denotes a statistically significant difference between treated and non-treated groups.

**Supplementary Table 11, related to Figure 6.** Number of macrophages, relative volume of collagen fibers and vessels in the structure of the glial scar in the spinal cord of DA rats treated by tocilizumab, infliximab, SB225002, MK-7123 and ТАК-779 60 days post SCI in comparison with non-treated animals. The asterisk denotes a statistically significant difference between treated and non-treated groups.

**Supplementary Table 12, related to Figure 6.** Count of GFAP-positive cells in a unit area of 500 x 500 μm on the border of the defect with intact tissue, average and maximum depth of its penetration into the structure of the glial scar of the spinal cord of DA rats treated by tocilizumab, infliximab, SB225002, MK-7123 and ТАК-779 60 days post SCI in comparison with non-treated animals. The asterisk denotes a statistically significant difference between treated and non-treated groups.

**Supplementary Video 1 related to Figure 1.** Computed tomography of the spine and surgical access site to the spinal cord.

**Supplementary Video 2, related to Figure 6.** Diffusion tensor imaging of injured area of intact DA rats on day 7 after SCI.

**Supplementary Video 3, related to Figure 6.** Diffusion tensor imaging of injured area of non-treated DA rats on day 7 after SCI.

**Supplementary Video 4, related to Figure 6.** Diffusion tensor imaging of injured area of DA rats treated by tocilizumab on day 7 after SCI.

**Supplementary Video 5, related to Figure 6.** Diffusion tensor imaging of injured area of DA rats treated by infliximab on day 7 after SCI.

**Supplementary Video 6, related to Figure 6.** Diffusion tensor imaging of injured area of DA rats treated by TAK-779 on day 7 after SCI.

**Supplementary Video 7, related to Figure 6.** Diffusion tensor imaging of injured area of DA rats treated by SB225002 on day 7 after SCI.

**Supplementary Video 8, related to Figure 6.** Diffusion tensor imaging of injured area of DA rats treated by MK-7123 on day 7 after SCI.

**Supplementary Table 1.** Count of GFAP-positive cells in a unit area of 500 x 500 μm on the border of the defect with intact tissue, average and maximum depth of its penetration into the structure of the glial scar of the spinal cord of SD rats at different time intervals after SCI

| Days post SCI | Average penetration depth of GFAP-positive cells, μm | Maximum penetration depth of GFAP-positive cells, μm | Average density of GFAP-positive cells | Maximum density of GFAP-positive cells |
| --- | --- | --- | --- | --- |
| 14 (n=6) | 271 ± 27 | 428 ± 35 | 8 ± 1 | 14 ± 2 |
| 30 (n=5) | 305 ± 23 | 455 ± 59 | 14 ± 2 | 27 ± 3 |
| 60 (n=6) | 342 ± 8 | 487 ± 27 | 20 ± 2 | 38 ± 5 |

**Supplementary Table 5.** Linear dimensions of the spinal cord defect (defect area, cranio-caudal and dorso-ventral dimensions) in SD rats treated by tocilizumab, infliximab and mogamulizumab 60 days post SCI in comparison with non-treated animals.

| Treatment | Defect area, mm^2^ | Cranio-caudal size of the defect, μm | Dorso-ventral dimension of the defect, μm |
| --- | --- | --- | --- |
| Placebo (n=6) | 2.8 ± 0.7 | 3.0 ± 0.6 | 1.9 ± 0.1 |
| Tocilizumab (n=5) | 3.4 ± 0.6 | 3.3 ± 0.6 | 2.1 ± 0.3 |
| Infliximab (n=10) | 3.0 ± 0.8 | 2.6 ± 0.4 | 1.7 ± 0.3 |
| Mogamulizumab (n=8) | 2.2 ± 0.3* | 2.5 ± 0.4 | 1.7 ± 0.2 |

**Supplementary Table 6.** Number of macrophages, relative volume of collagen fibers and vessels in the structure of the glial scar in the spinal cord of SD rats treated by tocilizumab, infliximab and mogamulizumab 60 days post SCI in comparison with non-treated animals. The asterisk denotes a statistically significant difference between treated and non-treated groups.

| Treatment | Macrophages count per unit area of the defect (37,500 µm^2^) | Relative volume of fiber component, mm^3^/mm^3^ | Relative volume of blood vessels, mm^3^/mm^3^ |
| --- | --- | --- | --- |
| Placebo (n=6) | 23 ± 2 | 0.122 ± 0.003 | 0.088 ± 0.003 |
| Tocilizumab (n=5) | 34 ± 6* | 0.058 ± 0.002* | 0.084 ± 0.002 |
| Infliximab (n=10) | 30 ± 8* | 0.24 ± 0.02* | 0.087 ± 0.002 |
| Mogamulizumab (n=8) | 38 ± 13* | 0.24 ± 0.01* | 0.084 ± 0.004 |

**Supplementary Table 7.** Size of cystic cavities in the structure of the glial scar in the spinal cord of SD rats treated by tocilizumab, infliximab and mogamulizumab 60 days post SCI in comparison with non-treated animals. The asterisk denotes a statistically significant difference between treated and non-treated groups. The asterisk denotes a statistically significant difference between treated and non-treated groups.

| Treatment | Maximum area of individual cystic cavities, mm^2^ | Total area of cystic cavities in the structure of glial scar, mm^2^ |
| --- | --- | --- |
| Placebo (n=6) | 0.4 ± 0.2 | 0.7 ± 0.2 |
| Tocilizumab (n=5) | 0.9 ± 0.4* | 1.7 ± 0.9* |
| Infliximab (n=10) | 0.6 ± 0.2 | 0.8 ± 0.2 |
| Mogamulizumab (n=8) | 0.4 ± 0.1 | 0.4 ± 0.1* |

**Supplementary Table 8.** Count of GFAP-positive cells in a unit area of 500 x 500 μm on the border of the defect with intact tissue, average and maximum depth of its penetration into the structure of the glial scar of the spinal cord of SD rats treated by tocilizumab, infliximab and mogamulizumab 60 days post SCI in comparison with non-treated animals.

| Treatment | Average penetration depth of GFAP-positive cells, μm | Maximum penetration depth of GFAP-positive cells, μm | Average density of GFAP-positive cells | Maximum density of GFAP-positive cells |
| --- | --- | --- | --- | --- |
| Placebo (n=6) | 342 ± 8 | 487 ± 27 | 20 ± 2 | 38 ± 5 |
| Tocilizumab (n=5) | 343 ± 21 | 534 ± 33 | 23 ± 2 | 36 ± 5 |
| Infliximab (n=10) | 382 ± 43 | 492 ± 40 | 21 ± 2 | 38 ± 3 |
| Mogamulizumab (n=8) | 341 ± 31 | 498 ± 28 | 20 ± 2 | 39 ± 3 |

**Supplementary Table 9.** Linear dimensions of the spinal cord defect (defect area, cranio-caudal and dorso-ventral dimensions) in DA rats treated by tocilizumab, infliximab, SB225002, MK-7123 and ТАК-779 60 days post SCI in comparison with non-treated animals.

| Treatment | Defect area, mm^2^ | Cranio-caudal size of the defect, μm | Dorso-ventral dimension of the defect, μm |
| --- | --- | --- | --- |
| Placebo (n=5) | 3.4 ± 1.9 | 3.6 ± 2.3 | 1.6 ± 0.3 |
| Infliximab (n=5) | 3.8 ± 0.8 | 4.0 ± 0.9 | 1.5 ± 0.1 |
| Tocilizumab (n=5) | 4.3 ± 0.6 | 4.3 ± 0.5 | 1.7 ± 0.3 |
| SB225002 (n=5) | 3.1 ± 0.2 | 3.3 ± 0.2 | 1.5 ± 0.2 |
| MK-7123 (n=5) | 3.4 ± 1.3 | 3.4 ± 0.7 | 1.7 ± 0.1 |
| ТАК-779 (n=5) | 3.8 ± 1.7 | 3.5 ± 0.6 | 1.6 ± 0.4 |

**Supplementary Table 10.** Size of cystic cavities in the structure of the glial scar in the spinal cord of DA rats treated by tocilizumab, infliximab, SB225002, MK-7123 and ТАК-779 60 days post SCI in comparison with non-treated animals. The asterisk denotes a statistically significant difference between treated and non-treated groups.

| Treatment | Maximum area of individual cystic cavities, mm^2^ | Total area of cystic cavities in the structure of glial scar, mm^2^ |
| --- | --- | --- |
| Placebo (n=5) | 0.5 ± 0.3 | 0.9 ± 0.8 |
| Infliximab (n=5) | 0.4 ± 0.2 | 0.9 ± 0.3 |
| Tocilizumab (n=5) | 0.7 ± 0.3 | 1.7 ± 0.6* |
| SB225002 (n=5) | 0.4 ± 0.3 | 0.6 ± 0.4 |
| MK-7123 (n=5) | 0.5 ± 0.3 | 0.8 ± 0.4 |
| ТАК-779 (n=5) | 0.6 ± 0.5 | 1.2 ± 0.5 |

**Supplementary Table 11.** Number of macrophages, relative volume of collagen fibers and vessels in the structure of the glial scar in the spinal cord of DA rats treated by tocilizumab, infliximab, SB225002, MK-7123 and ТАК-779 60 days post SCI in comparison with non-treated animals. The asterisk denotes a statistically significant difference between treated and non-treated groups.

| Treatment | Macrophages count per unit area of the defect (37,500 µm^2^) | Relative volume of fiber component, mm^3^/mm^3^ | Relative volume of blood vessels, mm^3^/mm^3^ |
| --- | --- | --- | --- |
| Placebo (n=5) | 23 ± 2 | 0.20 ± 0.04 | 0.086 ± 0.004 |
| Infliximab (n=5) | 29 ± 3* | 0.27 ± 0.05* | 0.0876 ± 0.0006 |
| Tocilizumab (n=5) | 33 ± 4* | 0.14 ± 0.03* | 0.087 ±0.002 |
| SB225002 (n=5) | 25 ± 9 | 0.16 ± 0.04 | 0.087 ± 0.001 |
| MK-7123 (n=5) | 25 ± 5 | 0.18 ± 0.04 | 0.088 ± 0.002 |
| ТАК-779 (n=5) | 27 ± 4 | 0.16 ± 0.04 | 0.085 ± 0.005 |

**Supplementary Table 12.** Count of GFAP-positive cells in a unit area of 500 x 500 μm on the border of the defect with intact tissue, average and maximum depth of its penetration into the structure of the glial scar of the spinal cord of DA rats treated by tocilizumab, infliximab, SB225002, MK-7123 and ТАК-779 60 days post SCI in comparison with non-treated animals. The asterisk denotes a statistically significant difference between treated and non-treated groups.

| Treatment | Average penetration depth of GFAP-positive cells, μm | Maximum penetration depth of GFAP-positive cells, μm | Average density of GFAP-positive cells | Maximum density of GFAP-positive cells |
| --- | --- | --- | --- | --- |
| Placebo (n=5) | 436 ± 15 | 535 ± 23 | 32 ± 1 | 40 ± 2 |
| Infliximab (n=5) | 538 ± 99 | 629 ± 50* | 31 ± 8 | 37 ± 9 |
| Tocilizumab (n=5) | 511 ± 31* | 587 ± 38 | 39 ± 4 | 43 ± 3 |
| SB225002 (n=5) | 514 ± 7* | 568 ± 26 | 33 ± 5 | 38 ± 3 |
| MK-7123 (n=5) | 487 ± 52 | 523 ± 42 | 35 ± 8 | 40 ± 6 |
| ТАК-779 (n=5) | 511 ± 27* | 666 ± 78* | 29 ± 1 | 38 ± 2 |
