# Supplementary material for: Repurposing of Chemokine Antagonists for Combined Phase‐Resolved Spinal Cord Injury Treatment
Source: Adv Sci (Weinh). 2025 Oct 28;13(1):e16569. doi: 10.1002/advs.202516569 (PMC12767007; doi:10.1002/advs.202516569)
Supplement: Supplementary file 2 — Supplemental Figure 1 [file ADVS-13-e16569-s008.pdf]

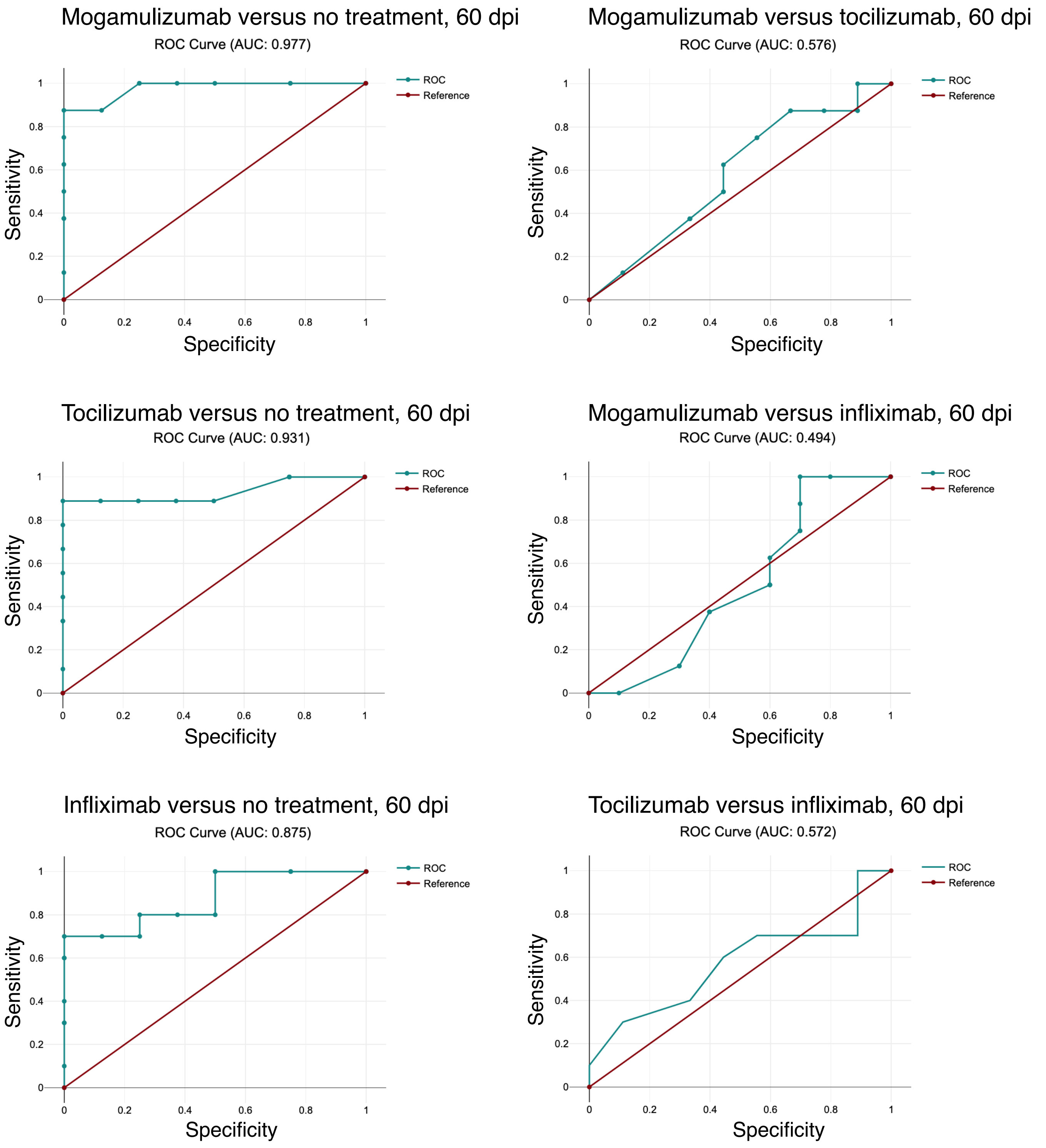

Supplementary Figure 1, related to Figure 5. Receiver operating characteristic area under the curve (AUC ROC) of BBB scores of treated and non-treated rats as indicated 60 days post injury.
