# Supplementary material for: Repurposing of Chemokine Antagonists for Combined Phase‐Resolved Spinal Cord Injury Treatment
Source: Adv Sci (Weinh). 2025 Oct 28;13(1):e16569. doi: 10.1002/advs.202516569 (PMC12767007; doi:10.1002/advs.202516569)
Supplement: Supplementary file 3 — Supplemental Figure 2 [file ADVS-13-e16569-s009.pdf]

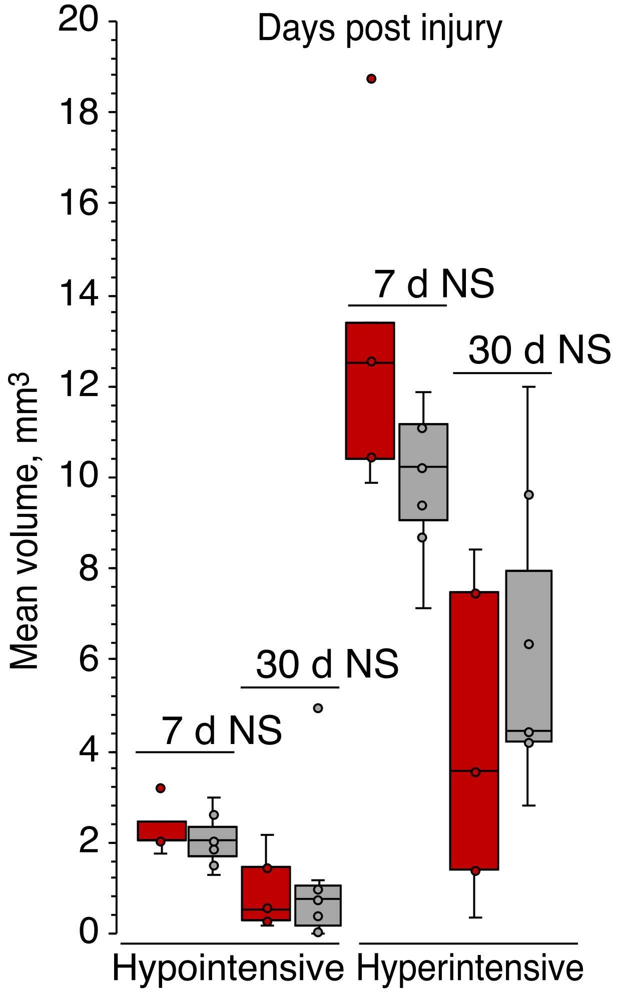

Supplementary Figure 2, related to Figure 6. Mean area of hypointensive and hyperintensive lesions in spinal cord of rats treated by dexamethasone (grey) and untreated rats (red) with SCI. Bars represent median, interquartile range and data spread. NS – non significant.
